# Female factors modulate Sex Peptide’s association with sperm in *Drosophila melanogaster*

**DOI:** 10.1186/s12915-022-01465-2

**Published:** 2022-12-14

**Authors:** Snigdha Misra, Norene A. Buehner, Akanksha Singh, Mariana F. Wolfner

**Affiliations:** 1 Department of Molecular Biology and Genetics, Cornell University, Ithaca, NY 14853 USA; 2 Present address: School of Health Sciences and Technology, University of Petroleum and Energy Studies, Dehradun, UK 248007 India; 3 Present address: Centre for Life Sciences, Mahindra University, Hyderabad, Telangana 500043 India

**Keywords:** *Drosophila*, Sex Peptide, Sperm, Seminal proteins, Sperm storage, Female reproductive tract, Parovaria, Spermathecal secretory cells

## Abstract

**Background:**

Male-derived seminal fluid proteins (SFPs) that enter female fruitflies during mating induce a myriad of physiological and behavioral changes, optimizing fertility of the mating pair. Some post-mating changes in female *Drosophila melanogaster* persist for ~10–14 days. Their long-term persistence is because the seminal protein that induces these particular changes, the Sex Peptide (SP), is retained long term in females by binding to sperm, with gradual release of its active domain from sperm. Several other “long-term response SFPs” (LTR-SFPs) “prime” the binding of SP to sperm. Whether female factors play a role in this process is unknown, though it is important to study both sexes for a comprehensive physiological understanding of SFP/sperm interactions and for consideration in models of sexual conflict.

**Results:**

We report here that sperm in male ejaculates bind SP more weakly than sperm that have entered females. Moreover, we show that the amount of SP, and other SFPs, bound to sperm increases with time and transit of individual seminal proteins within the female reproductive tract (FRT). Thus, female contributions are needed for maximal and appropriate binding of SP, and other SFPs, to sperm. Towards understanding the source of female molecular contributions, we ablated spermathecal secretory cells (SSCs) and/or parovaria (female accessory glands), which contribute secretory proteins to the FRT. We found no dramatic change in the initial levels of SP bound to sperm stored in mated females with ablated or defective SSCs and/or parovaria, indicating that female molecules that facilitate the binding of SP to sperm are not uniquely derived from SSCs and parovaria. However, we observed higher levels of SP (and sperm) retention long term in females whose SSCs and parovaria had been ablated, indicating secretions from these female tissues are necessary for the gradual release of Sex Peptide’s active region from stored sperm.

**Conclusion:**

This study reveals that the SP-sperm binding pathway is not entirely male-derived and that female contributions are needed to regulate the levels of SP associated with sperm stored in their storage sites.

**Supplementary Information:**

The online version contains supplementary material available at 10.1186/s12915-022-01465-2.

## Background

Molecular interactions between the male’s seminal fluid proteins (SFPs), sperm, and the female’s reproductive tract (FRT) are fundamental to successful reproduction [1–4]. For example, in *Drosophila melanogaster*, SFPs derived from glandular tissues of the male’s reproductive tract induce egg production, behavioral changes [1, 5], and physiological changes (e.g., [6]) including ovulation (ovulin [7]) and/or participate in the formation of the mating plug (Acp36DE, pEBme, pEBII [8–10]) within the reproductive tract, as well as having a variety of other systemic effects [11–15]. In addition, several *Drosophila* SFPs associate with sperm [4, 16, 17]. One sperm-associated SFP, Sex Peptide (SP), is retained in the sperm storage organs of female reproductive tract (the spermathecae [ST] and seminal receptacle [SR]) long term due to its association with sperm [18]. SP’s active C-terminal region is gradually released from sperm by trypsin cleavage [18] and induces long-term post-mating responses such as increased egg production and decreased receptivity to remating [5, 19–21].

Previous studies have shown that SP’s binding to sperm requires several other SFPs, acting in a network (the “long-term response (LTR) network” [3, 4]). Two SFPs in this network (Seminase, CG17575 [4, 22]) facilitate the binding of other SFPs to sperm, but do not themselves bind sperm. Other SFPs in the network (CG1656 [lectin-46Ca], CG1652 [lectin-46Cb], CG9997, Antares [16, 23]) bind to sperm transiently; their action is thought to “prime” sperm and/or SP to retain SP on sperm [17]. Thus far all molecules known to promote SP binding to sperm have been male-derived SFPs. Although some female proteins, such as Fra mauro, Hadley, Esp, and sex peptide receptor (SPR) are known to be necessary for SP-induced post-mating responses, their action is downstream of the binding of SP to sperm [24–28].

Whether the female also contributes to the binding of SP to sperm is unknown, but several recent findings suggested the importance of testing this possibility. First, female molecules play roles in modification (cleavage) of some SFPs in *D. melanogaster* [29], and in the proteolytic dissolution of the mating plug in cabbage-white butterflies, *Pieris rapae* [30], indicating that FRT proteins can have direct effects on SFPs and their molecular milieu. Second, active involvement by females in relative paternity proportions following mating with two males suggests that female molecules or cells can interact with ejaculate components (at least, sperm) [31–33]. Third, *Drosophila* sperm interact with proteins synthesized by the FRT after their transfer and prior to their storage in the female’s sperm storage (spermatheca (ST) and seminal receptacle (SR)) [34, 35]. Although we do not know the extent to which (or how) these female molecules might contribute to the interaction of sperm and SFPs, the molecular composition of sperm is known to change within the mated female due to association of multiple female-derived proteins with sperm [36]. Female contributions to SP’s binding to sperm could involve such known FRT molecules [28, 37–39] or ones as-yet unidentified.

Here, we examined the spatial and temporal characteristics of the association of SP and of the LTR- SFPs with sperm before and after their transfer to the FRT. We show that levels of sperm-bound SFPs are weak or undetectable in ejaculate collected from males, but that sperm binding by SFPs, including SP, becomes detectable (or increases) after the male ejaculate enters the female. The pattern and the signal intensity of binding of individual SFPs to sperm differ temporally and spatially within the FRT. This increase in their signal intensity level indicates that female components must play a role in priming sperm and/or SFPs to bind each other. To identify such female components, we disabled two secretory tissues in the FRT (spermathecal secretory cells (SSCs) and parovaria (female accessory glands) [37, 40, 41]) using *Hr39* mutations. We did not observe any dramatic effects in initial binding of SP to sperm stored in the mutant females suggesting that female molecules that assist in the binding of SP to sperm come from tissues other than, or in addition to, SSCs and parovaria. However, our data pinpointed a role for female secretions from SSCs and parovaria in a later interaction between SP and sperm: specifically, in the gradual release of sperm-associated SP that is important in long-term persistence of its effects on females.

Our finding that females, as well as males, contribute molecules needed to bind SFPs to sperm and to cleave SP’s active region from sperm has implications for understanding the molecular cooperation between the sexes that leads to optimal fertility of the mating pair, as well as for models of sexual conflict, and motivates future studies to identify the specific female molecules that assist in binding SP to sperm, or that mediate its release from sperm.

## Results

### Sex Peptide binds sperm weakly in the male ejaculate but its binding increases within the mated female’s reproductive tract

To test whether female factor(s) affect the binding of SP to sperm, we compared the signal intensity of anti-SP staining on sperm before (in ejaculate collected from males) and after mating (in the female’s bursa [uterus] and SR). We reasoned that if the signal intensity in the male ejaculate did not change after mating, this would mean that components of the male ejaculate are sufficient to fully facilitate SP-sperm binding without requiring female factor(s).

**Fig. 1:**
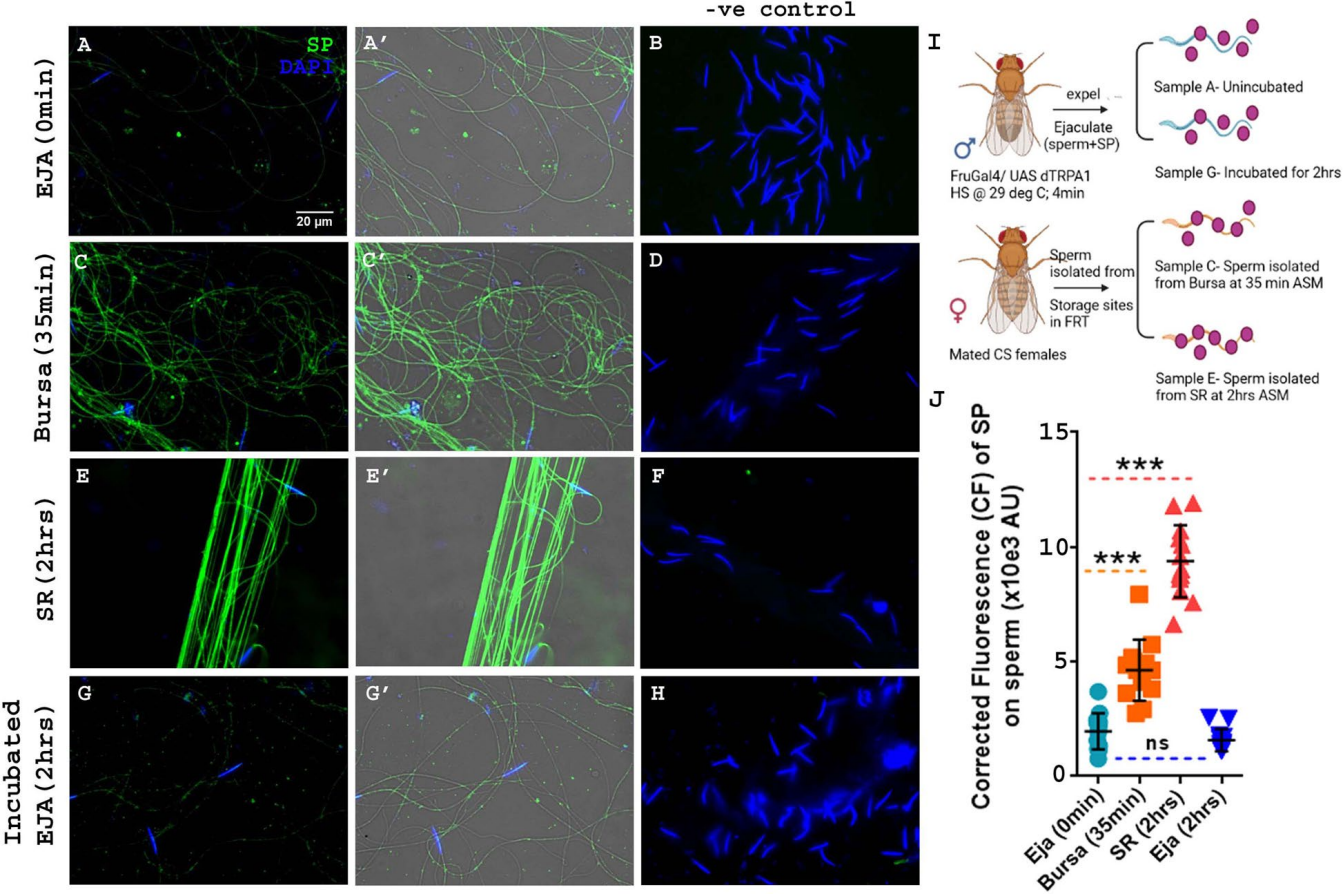
Amount of SP detected on sperm gradually increases from male ejaculate to female bursa and is highest in seminal receptacles. Pre-mating ejaculate samples were collected from *Fru>dTRPA1* males exposed to high temperatures, as described in “Methods”. Post-mating sperm samples were isolated from wild type (CS) females that had mated to wildtype (CS) males and were frozen at 35 min and 2 h ASM. Sperm heads were stained with DAPI (blue) and anti-SP staining was visualized with Alexa fluor 488, staining the sperm tail (green) and sperm head (cyan; overlapping blue/green); Bar = 20μm. **A, A’** Sperm isolated from ejaculate expelled by Fru>dTRPA1 males. **C, C’** Sperm isolated from bursa of the wildtype mated female, frozen at 35 min ASM. **E, E’** Sperm isolated from the seminal receptacle of the wildtype mated female, frozen at 2 h ASM. **G, G’** Sperm isolated from Fru>dTRPA1 male’s ejaculate that was kept incubated for 2 h in 1× PBS, after exudation. **B, D**, **F**, and **H** are negative controls for **A**, **C**, **E**, and **G** panels, with only secondary antibody (anti- rabbit, Alexa fluor 488) and no primary antibody (anti-SP) incubation. **A’**, **C’**, **E’**, and **G’** panels have an additional transmitted light channel overlay to highlight the outline of sperm tail specifically in samples where the staining was very weak (ejaculate). **I** Schematic of the time and nature of sample collection. **J** Corrected fluorescence (CF) intensity of SP on sperm at three different stages and time points. Error bars show Mean±SE (AU stands for arbitrary units); p***<0.001, p**<0.01, ns=not significant, degree of freedom, F(3, 48)= 132; *n*=13

We isolated sperm from ejaculates exuded by males (Eja; 0 min), sperm in the mated female’s bursa (uterus; 35 min after the start of mating; ASM) or stored in her seminal receptacle (SR; 2 h ASM). The amount of SP bound to sperm was determined by quantifying the corrected signal intensity of the immunofluorescence of anti-SP along the sperm tail in all three situations. The signal intensity for SP detected on sperm was weakest in male ejaculates (Fig. 1A, A’, and J; Mean±SE=1964±442.6 AU; F(3, 48) = 132). It was higher in sperm isolated from mated female bursas. Sperm isolated from mated female bursas (35 min ASM) had a “spotty” pattern of anti-SP staining, with anti-SP immunofluorescence appearing in bright and dim specks all along sperm (Fig. 1C, C’, and J, Mean±SE=4361±442.6 AU, p***<0.001; compare to Fig. 1A, A’). This suggested that although the quantity of SP bound to sperm increased in the female’s bursa, sperm were not uniformly saturated with SP. Sperm isolated from the SRs (2 h ASM) had the strongest signal intensity of SP, suggesting that the amount of SP detected on sperm was highest in the sperm storage organ. Staining for SP on sperm isolated from SRs was consistent and uniform along the sperm (Fig. 1E, E’, and J; Mean±SE=9384±442.6 AU, p***<0.001), similar to what has been reported previously [16–18]. Since the amount of SP detected on sperm gradually increases after they enter the FRT, our results suggest a possible role of female factor(s) in assisting SPs binding to stored sperm.

Several SFPs (proteases, prohormones, and others) either mediate or undergo post-mating modifications en route to or after transfer to the FRT [7, 29, 42], some of which are crucial for inducing or maintaining post-mating responses in mated females. We thus wondered whether the gradual increase in amount of SP detected on sperm within the FRT is because of a need for the male components to undergo requisite modifications with time. The intensity of SP signals on sperm was observed to be highest in sperm isolated from the SR at 2 h ASM, suggesting that this is the maximum time that would be required by the male molecules to act (or to undergo any necessary modifications). To test if time alone is sufficient to maximize SP’s binding to sperm, we collected ejaculates exuded from males and incubated them for 2 h in 1× PBS before processing them for anti-SP staining. We did not observe any change in the signal intensity or in the distribution of anti-SP on sperm (Fig. 1G, G’, and J, Mean±SE=1578±442.6 AU, *p*=ns) in incubated ejaculates relative to signals on sperm isolated from un-incubated ejaculates (Fig. 1A, A’, and J). This suggested that time alone is not sufficient to maximize SP’s binding to sperm. Thus, female factor(s) likely contribute to, or facilitate, SP-sperm binding. As males with different genetic backgrounds and exposure to temperature conditions were used in these experiments, we verified that these differences did not affect the levels of SP that we observed to be associated with sperm stored in the SR of mated females (Please see Additional file 1: Fig. S1 for details).

### LTR-SFPs bind to sperm in the male’s ejaculate or mated females with patterns or timing different from those of SP

Given LTR-SFPs’ role in SP’s sperm binding, we wondered whether the pattern of sperm-associated CG1656, CG1652, CG9997, and Antares (Antr) on sperm isolated from three different sites/times used above paralleled that of SP. We examined the presence of bound LTR-SFPs to sperm by experiments analogous to those shown in Fig. 1 for SP, using sperm isolated from the male’s ejaculate (0 min after exudation), mated female’s bursa (35 min ASM), and SR (2 h ASM).

**Fig. 2:**
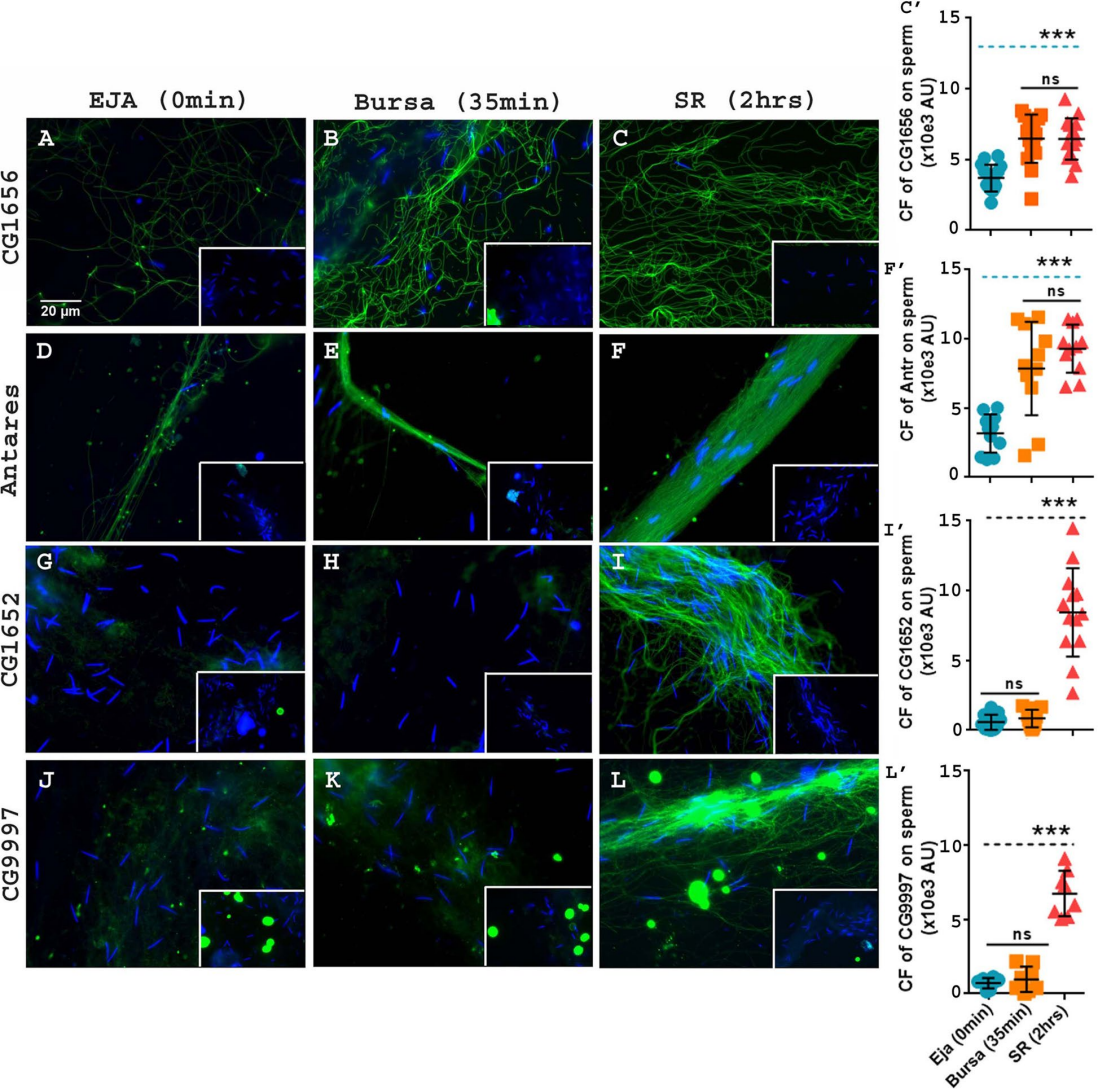
The levels of LTR-SFPs bound to sperm increase from male ejaculate to sperm stored in female seminal receptacle, but the pattern differs from SP’s. Pre-mating ejaculate samples were collected from *Fru>dTRPA1* males exposed to high temperatures, as described in “Methods”. Post-mating sperm samples were isolated from wild type (CS) females that had mated to wildtype (CS) males and frozen at 35 min and 2 h ASM. Sperm heads were stained with DAPI (blue) and LTR-SFPs were visualized with Alexa fluor 488, staining the sperm (green); Bar = 20μm. Sperm isolated from male ejaculate immediately after exudation (**A**, **D**, **G**, and **J**) were probed for CG1656 (degree of freedom, F(2, 42)=19.48), Antares (F(2,30)=20.77), CG1652 (F(2,36)=72.79), and CG9997 (F(2,21)=87.66), respectively. Sperm isolated from mated female bursa, frozen at 35 min ASM (**B**, **E**, **H**, and **K**) were probed for CG1656, Antares, CG1652, and CG9997, respectively. Sperm isolated from mated female’s seminal receptacle, frozen at 2 h ASM (**C**, **F**, **I**, and **L**) were probed for CG1656, Antares, CG1652, and CG9997, respectively. The insets show the negative controls for their respective panels. Sperm samples in negative controls were incubated with only secondary antibody (anti-rabbit, Alexa fluor 488) but no primary antibody (anti-LTR-SFP) incubation, as mentioned previously. **C’**, **F’**, **I’**, and **L’** show corrected fluorescence (CF) intensity of CG1656, Antares, CG1652, and CG9997 on sperm at three different stages and time points. Error bars show Mean±SE (AU stands for arbitrary units); p***<0.001, ns=not significant, *n*=8–15

We observed a lower signal intensity for CG1656 (Fig. 2A, C’; Mean±SE=3689±513.3 AU; F(2, 42)=19.48) and Antr (Fig. 2D, F’; Mean±SE=3173±993.5 AU; F(2,30)=20.77) on sperm in ejaculate compared to that on sperm inside the female (Fig. 2B (6474±513.3 AU; p***<0.001), C (6453±513.3AU; p***<0.001) and C’ for CG1656 and E (7858±993.5 AU; p***<0.001), F (9296±993.5 AU; p***<0.001), and F’ for Antr. However, the signal intensity of sperm-bound CG1656 and Antr did not differ between sperm isolated from the bursa (Fig. 2B, E) vs. those from the SRs (Fig. 2C, F). This suggests that although the amount of these LTR-SFPs bound to sperm increases post-mating, their maximal binding had already occurred in the bursa of the mated female, in contrast to SP whose sperm binding reached its highest levels in the female’s SR.

CG1652 and CG9997 differed in their sperm-binding pattern from CG1656 and Antr. We detected extremely faint signals for both CG1652 and CG9997 associated with sperm in the ejaculate (Fig. 2G, I’ (Mean±SE=576±740.3 AU; F(2,36)=72.79), Fig. 2J, L’ (Mean±SE=715.5±515.4 AU; F(2,21)=87.66) respectively) or in the bursa of the mated female (Fig. 2H, I’ (Mean±SE=838±740.3 AU; *p*=ns), Fig. 2K, L’ (Mean±SE=968.4±515.4 AU; *p*=ns), respectively). However, we saw significantly strong signal for both proteins in sperm isolated from SRs of mated females (Fig. 2I, I’ (Mean±SE=8439±740.3 AU; p***<0.001), 2L, L’ (Mean±SE=6748±515.4 AU; p***<0.001) respectively), consistent with our previous report that these proteins are bound to sperm in SRs [4, 16]. The regions of association and distribution that we observed for these SFPs (SP, CG9997, and Antr on the head and tail of stored sperm; CG1652 and CG1656 detectable only on the tail of stored sperm) were also consistent with previous reports that assessed the levels of SP associated with sperm stored in SRs [4, 16].

We also assessed the two LTR-SFPs, Seminase and CG17575, that had previously been reported as not binding to stored sperm [4, 22]; in addition to confirming that finding, our experiments showed that these two SFPs exhibit no sperm binding in the ejaculate either (ejaculate: Additional file 2: Fig. S2A and D; mated female’s, bursa: Additional file 2: Fig. S2B and E; SR: Additional file 2: Fig. S2C and F). Thus, the binding patterns/timing of LTR-SFPs differed from those of SP and fall into three groups: (1) CG1656 and Antr, which bind to sperm in the ejaculate, increase their binding once inside the female, but do not show the additional increase in binding in the seminal receptacle that was seen for SP; (2) CG1652 and CG9997 show no detectable binding to sperm until they are inside the female’s seminal receptacle; (3) Seminase and CG17575 show no detectable binding to sperm.

### Ablation of spermathecal secretory cells (SSCs) in the female reproductive tract does not affect the initial binding of SP or LTR-SFPs to sperm

**Fig. 3:**
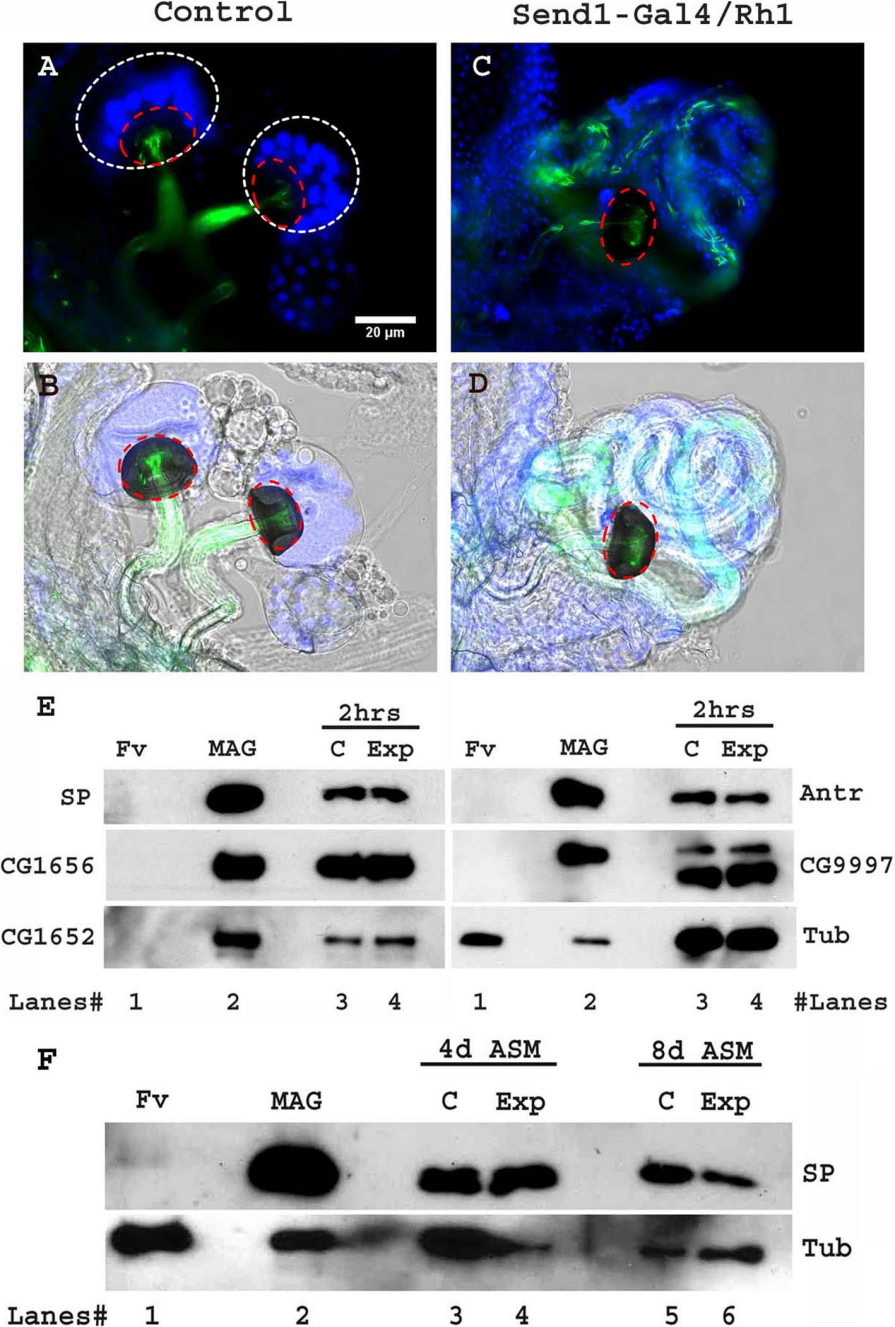
Ablated SSCs in *Send1>Rh1*^*G69D*^ females affects neither the binding of SP to sperm (at 2 h ASM) nor the long-term gradual cleavage of SP from sperm (at 4 days or 8 days ASM). Expression of ER stress-inducing Rh1^G69D^ by spermathecae-specific Send1-Gal4 results in ablation of SSCs. **A,B** Control (*Send1>CyO*) females mated with *ProtB-eGFP* males (eGFP-tagged sperm; green) show normal numbers of SSCs (stained with DAPI; white dotted circle) lining the spermathecal cap (red dotted circle). **C,D** Experimental (*Send1>Rh1*^*G69D*^) females mated with *ProtB-eGFP* males show ablated SSCs (stained with DAPI) lining the spermathecal cap (red dotted circle); *n*=10; Bar = 20μm. **E** Western blot probed for SP and indicated LTR- SFPs at 2 h ASM. *Lanes# 1*: Fv, reproductive tract (RT) of 4 unmated females (negative control), *2*: MAG, 1 pair of male accessory glands (positive control), *3*: C, sperm dissected from SR of 30 control (*Send1>CyO*) females mated to wild type (CS) males at 2 h ASM, *4*: Exp, sperm dissected from SR of 30 experimental (*Send1>Rh1*^*G69D*^) females mated to wild type (CS) males at 2 h ASM. Lanes were probed for SP and LTR-SFPs CG1656, CG1652, Antares, and CG9997 as described in the text. **F** Western blot probed for SP at 4 and 8 days ASM. *Lanes# 1*: Fv, reproductive tract (RT) of 4 unmated females (negative control), *2*: MAG, 1 pair of male accessory glands (positive control), *3*: C, sperm dissected from SR of 30 control (*Send1>CyO*) females mated to wild type (CS) males at 4 days ASM, *4*: Exp, sperm dissected from SR of 30 experimental (*Send1>Rh1*^*G69D*^) females mated to wild type (CS) males at 4 days ASM, *5*: C, sperm dissected from SR of 30 control (*Send1>CyO*) females mated to wild type (CS) males at 8 days ASM, *6*: Exp, sperm dissected from SR of 30 experimental (*Send1>Rh1*^*G69D*^) females mated to wild type (CS) males at 8 days ASM. Lanes were probed for SP. Tubulin served as loading control

To begin to identify the source of female contributions to SFP-sperm binding, we examined the effect on the intensity and timing of SFP binding to sperm when we ablated SSCs, which are known to regulate the storage and motility of sperm in sperm storage organs [37, 40, 41]. We ablated SSCs by driving the expression of misfolded protein Rh^G69D^ [43, 44] in these cells (Fig. 3A–D) or by using *Hr39* mutants (Please see Additional file 4: Fig. S4). Hr39, a NR5A-class nuclear hormone receptor, is needed for the development and function of important secretory tissues in the FRT [37]: *Hr39* mutants exhibit defective/decreased SSCs and parovaria [41, 45]. Although neither targeting of *Rh1*^*G69*D^ to SSCs nor the presence of *Hr39* mutations completely ablated all SSCs, higher percentages of ablations were observed in *Hr39* mutant females. We examined SP-sperm binding in these SSC-depleted females.

Five-day-old *Send1>CyO* (control) and *Send1>Rh1*^*G69D*^ (experimental) females were mated to 3-day-old control (CS) males, and the mated females were frozen at 2 h ASM. Sperm, dissected from SRs of the mated females, were assessed for the presence of SP by western blotting. We did not observe any striking difference in the levels of sperm-associated SP in experimental females relative to control females at 2 h ASM (Fig. 3E, lanes 3, 4). Similarly, there was no difference in amounts of LTR-SFPs CG1656, CG1652, Antr, or CG9997 associated with sperm isolated from experimental vs. control females at 2 h ASM (Fig. 3E, lanes 3, 4). We also performed immunofluorescence (IF) on sperm dissected from the SR of *Send1>CyO* (control) and *Send1>Rh1*^*G69D*^ (experimental) females to probe for the presence of SP. Consistent with the results of our western blots, we did not observe any striking difference in the intensity of anti-SP staining on sperm stored in experimental females (Additional file 3: Fig. S3B-B’ and E; Mean±SE=4299±224.8 AU; F(10, 10)=2.023; *p*=0.2818) relative to those stored in control females (Additional file 3: Fig. S3A-A’ and E; Mean±SE=4590±319.8 AU) at 2 h ASM.

**Fig. 4:**
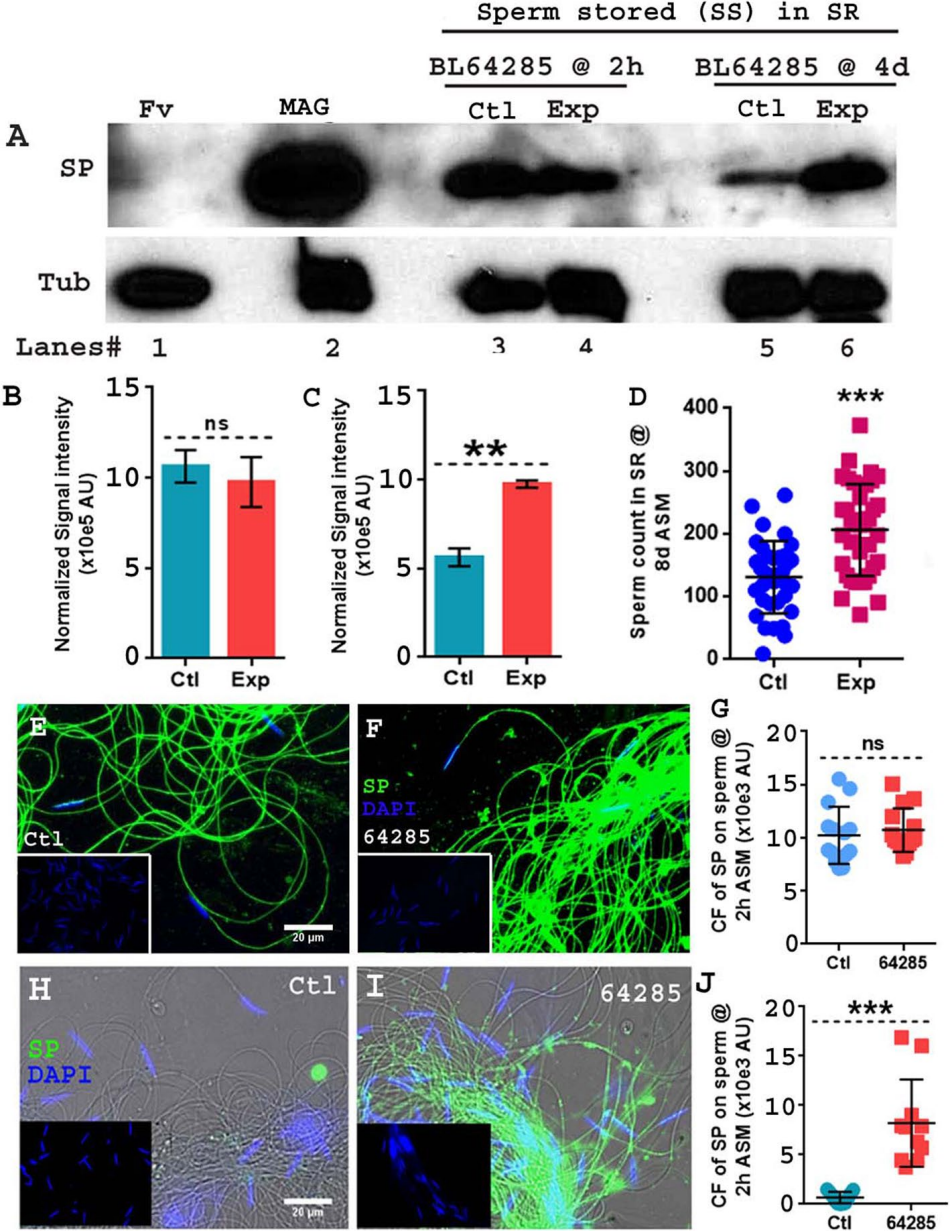
Loss of SSCs and/or parovaria in *Hr39* mutant females does not inhibit the binding of SP to sperm but leads to retention of sperm and therefore elevated SP levels long term. **A** Western blot probed for SP. *Lanes# 1*: Fv, reproductive tract (RT) of 4 unmated females (negative control), *2*: MAG, 1 pair of male accessory glands (positive control), *3*: Ctl, sperm dissected from SR of 30 genetically matched control (BL64285) females mated to wild type (CS) males at 2 h ASM, *4*: Exp, sperm dissected from SR of 30 mutant (BL64285) females mated to wild type (CS) males at 2 h ASM, *5*: Ctl, sperm dissected from SR of 30 genetically matched control (BL64285) females mated to wild type (CS) males at 4 days ASM, *6*: Exp, sperm dissected from SR of 30 mutant (BL64285) females mated to wild type (CS) males at 4 days ASM. Tubulin (Tub) served as loading control. **B** Graphical representation of the normalized levels of sperm-bound SP in *Hr39* mutant (exp; pink bar) females relative to genetically matched control females (Ctl; blue bar) from the stock BL64285, at 2 h ASM (ns= nonsignificant; *p*=0.5328; df=2). **C** Graphical representation of the normalized levels of sperm-bound SP in *Hr39* mutant (exp; pink bar) females relative to genetically matched control females (C; dark blue bar & dotted line) from the stock BL64285, at 4 days ASM (p**<0.01; df=2; AU stands for Arbitrary Units ). **D** Graphical representation of sperm counts in SRs of genetically matched control (C; blue bar) and *Hr39* mutant (Exp; pink bar) females from BL64285 stocks mated to control *ProtB-eGFP* males (with eGFP-tagged sperm; p***=<0.001; *n*=15–20) and frozen at 8 days ASM. Error bars show Mean±SE. Sperm samples isolated from the seminal receptacle of **E** matched control females, **F** BL64285, *Hr39* mutant females*.* The females were mated with CS males and frozen at 2 h ASM. **G** Corrected fluorescence (CF) intensity of SP bound to stored sperm in matched control (Ctl) and BL64285 females at 2 h ASM performed through immunofluorescence. Error bars show Mean±SE AU, F(13,13)=1.729, *p*=0.3360, ns= nonsignificant; *n*=14. Sperm samples isolated from the seminal receptacle of matched control females (**H**), BL64285, *Hr39* mutant females (**I**). **J** Corrected fluorescence (CF) intensity of SP bound to stored sperm in matched control (Ctl) and BL64285 females at 4 days ASM performed through immunofluorescence. Error bars show Mean±SE AU, F(10,10)=60.63, p***<0.001; *n*=11. In all the immunofluorescence panels, sperm heads were stained with DAPI (blue) and anti-SP staining was visualized with Alexa fluor 488, staining the sperm tail (green) and sperm head (cyan; overlapping blue/green). The insets show the respective negative controls each panel. The bigger panels (**H** and **I**) in the 4day samples have transmitted light filter added to show the outline of sperm tail in the regions where SP was lowly detected (e.g., panel **J**); Bar = 20μm

In similar experiments, 5-day-old *Hr39* mutant and control females were mated to 3-day-old control (CS) males, and mated females were frozen at 2 h ASM. We dissected sperm from SRs of *Hr39* mutant (BL64285 {*Hr39[C105]*}; Exp) [46] and their balanced sibling (“sib”) controls (BL64285/*CyO*; C) at 2 h ASM and probed the samples for SP by western blotting and immunofluorescence. As we saw with *Send1>Rh1*^*G69D*^ vs. control females, we did not observe any striking difference in the levels of SP associated with sperm probed through western blotting (Fig. 4A, lanes 3, 4; Fig. 4B; *p*= 0.5328) or the signal intensity of anti-SP staining along the entire sperm performed through immunofluorescence in control balancer-sib females (Fig. 4E, G; Mean±SE=10238±715.9 AU; F(13, 13)=1.729; *p*=0.3360; *N*=14) when compared to mutant females at 2 h ASM (Fig. 4F, G; Mean±SE=10736±544.5 AU; *N*=14). To test for consistency, we examined four other available *Hr39* mutants. However, three of these lines (BL38620 {*Hr39[MI06174]*} [47], BL43358 {*Hr39[C277]*} [48], and BL20152 {*Hr39[EY04579]*} [49]) did not contain balancer-sib controls, and the fourth (BL64305/CyO {*Hr39[c739]*} [50]) produced too few such balancer-sib females for experimentation, so we used *CS* females as their controls. We performed western blotting to examine the effect of differences in the genetic background for two different controls (balancer-sib and *CS* females) used in our experiments on the levels of SP received by females. We observed no striking difference in the levels of SP transferred to the FRT at 35 min ASM (Additional file 8: Fig. S8 lanes 3 and 4) and those bound to sperm stored in the SR at 2 h ASM (Additional file 8: Fig. S8 lanes 5 and 6) in both controls. As we saw with BL64285 females and their controls, we detected similar levels of SP bound to sperm dissected from *CS* and these *Hr39* mutant females (Additional file 5: Fig. S5A, lane 3, lanes 4–8; Additional file 5: Fig. S5B) at 2 h ASM. We also detected signals for the LTR-SFPs CG1656, CG1652, Antr, and CG9997 in *Hr39* mutant females at levels similar to those in *CS* females, at 2h ASM (Additional file 6: Fig. S6A, lane 3 and lanes 4–8). The level of anti-SP staining visualized along the entire sperm through immunofluorescence also did not show any relative difference between *CS* females (control) (Additional file 5: Fig. S5C & H) and mutant females from the other four *Hr39* lines (Additional file 5: Fig. S5D-H), consistent with what we observed in our western blots. Thus, loss of SSCs (and parovaria, in the case of *Hr39* mutants) did not have an evident effect on the binding of SP or LTR-SFPs to sperm. This result suggested that female molecules that increase the levels of SFPs and SP association with stored sperm are not derived (or not solely derived) from SSCs and/or parovaria.

### Loss of SSCs and parovaria in females affects the release of SP from stored sperm

At 4 days ASM, our western blots consistently showed higher levels of sperm-associated SP in *Hr39* mutant females (BL 64285; Fig. 4A, lane 6) relative to the levels in their balancer-sib control females (Fig. 4A, lane 5); this was particularly clear when SP levels were normalized with tubulin levels (Fig. 4C; p**<0.01). We obtained analogous results showing higher levels of sperm-associated SP at 4 days ASM in females homozygous for each of the other four *Hr39* mutant alleles, relative to sperm-bound SP levels in *CS* females (Additional file 7: Fig. S7A lanes, 3–8), following normalization of SP levels with tubulin (Additional file 7: Fig. S7B).

Binding of SP to sperm or SP’s gradual cleavage from sperm are both essential for the efficient release of sperm from storage within the mated female [51]. To test whether the elevated levels of sperm-bound SP in *Hr39* mutant females at 4d ASM was associated with increased retention of sperm in these females, we performed sperm counts. Control and *Hr39* mutant females from the BL64285 stock were mated to *ProtB-eGFP* [52] males, and sperm stored in their SR were counted at 8 days ASM. Mutant females (Fig. 4D, Exp) exhibited significantly higher sperm numbers relative to their controls (Fig. 4D, C; p**=<0.01). Consistently, mated *Hr39* mutant females from the other four lines also showed significantly higher sperm counts, indicating poor release of stored sperm when compared to *CS* females (Additional file 6: Fig. S6B; p**=<0.01).

To distinguish whether the higher amounts of sperm-associated SP measured on western blots were due to this higher retention of sperm or to impaired release of SP from sperm, we used immunofluorescence to examine levels of SP bound to sperm. We observed higher levels of sperm-bound SP in BL64285 *Hr39* mutant females (Fig. 4I, J; Mean±SE=8209±1332 AU;F(10, 10)=60.6; p**<0.001; *N*=11) than in balancer-sib control females (Fig. 4H, J; Mean±SE=673.5±171.1 AU); the SP levels in balancer-sib controls at 4 days ASM were either barely detectable or below our detection limits. Similarly, anti-SP immunofluorescence was higher in females for the four other *Hr39* mutants (Additional file 7: Fig. S7D-H) relative to levels in *CS* controls (Additional file 7: Fig. S7C & H); as observed with balancer-sib controls for BL64285, the *CS* controls again had SP levels barely detectable or below our detection limits at 4 days ASM. We used *Send1>Rh1*^*G69D*^ flies, in an attempt to determine whether SSCs alone were responsible for effects on SP release. We saw no difference in SP levels bound to sperm dissected from *Send1>CyO* (control) and *Send1>Rh1*^*G69D*^ (experimental; SSCs ablated) females mated to *CS* (control) males (Fig. 3F, lanes 3, 4 for 4 days ASM and 5, 6 for 8 days ASM) in our western blotting. The level of anti-SP staining visualized along the entire sperm through immunofluorescence also did not show any relative difference between experimental females (Additional file 3: Fig. S3D-D’ and F; Mean±SE=38.14±12.93 AU; F(6, 6)=2.1; *p*=0.3651) relative to those stored in control females (Additional file 3: Fig. S3C-C’ and F; Mean±SE=50.86±19.1 AU) at 4 days ASM. However, signals were extremely low in these experiments. Our results suggest that loss of some or all SSCs may not be sufficient to impair the release of SP from sperm, but that the absence of SSCs and parovaria together impairs the release of SP from sperm. Since the release of sperm from storage requires this release [51], the lack of SP release in the absence of SSCs and parovaria results in more sperm being retained in females (and each of these sperm contains more bound SP than would normally be seen at those times). These data suggest and support the hypothesis that the protease responsible for cleaving SP’s active region from sperm [18] is provided by the parovaria and/or SSCs.

## Discussion

In addition to their crucial role in fertilization, sperm can have functions that modulate other aspects of reproduction. For example, *Drosophila* sperm can bind SP (and several other SFPs), causing SP’s retention in the female and allowing it to induce long-term physiological and behavioral changes [3, 4, 16, 18]. Given that sperm can potentiate the effect of this male-derived protein, it is of interest to know whether the male, female, or both facilitate this binding. Previous studies showed that several SFPs are needed to bind SP to sperm, indicating male contributions to this phenomenon. Although previous studies have identified a few female proteins (Fra mauro, Hadley, Esp, and sex peptide receptor (SPR)) that are needed for SP activity, none of these proteins affected SP binding to sperm [24, 28, 39, 53]. Here, we show that female-derived factors are also necessary for SFPs, including SP, to bind to sperm and to release SP from sperm. We report that levels of sperm-bound SFPs and SP are weak to undetectable in the male’s ejaculate, but increase once the ejaculate is within the FRT. Incubation experiments show that this increase is not simply due to time, but requires that the ejaculate be within the female, pointing to the need for female contributions to the SFP-sperm binding. Although our cell-ablation experiments showed that secretions from the SSCs and parovaria are not uniquely necessary for this particular contribution to SP-sperm binding, we discovered that those cells’ secretions are necessary for the subsequent release of SP from sperm that is needed for its long-term activities, such as release of sperm from storage. Our results suggest a molecular cooperation between male and female in the regulation of the binding of SFPs to sperm.

### Levels of sperm-bound SP and LTR-SFPs increase within the mated female, though not all exhibit the same pattern

SP-sperm binding is regulated by a network of “LTR-SFPs” [4, 51] whose members include the cysteine-rich secretory proteins (CRISPs) CG17575 and Antr, the proteases Seminase and CG9997, and the lectins CG1656 and CG1652 [3, 4, 22]. LTR-SFPs exist transiently within the FRT and help prime or modify sperm to bind SP for the sustenance of long-term post-mating responses [16, 17, 36].

Interestingly, we observed differences in sperm binding by SP and individual LTR-SFPs with respect to timing and/or localization within the FRT. First, SP and two LTR-SFPs (CG1656 and Antr) were detected at low levels on sperm in ejaculate; the other LTR-SFPs were not detectable on sperm in ejaculate. Second, once ejaculate entered the female’s bursa, levels of SP, Antr, and CG1656 increased on sperm, but the other LTR-SFPs remained undetectable on sperm. Third, once sperm entered the SR, levels of CG9997 and CG1652 and increased levels of SP were detected on sperm. While levels of anti-SP staining on sperm increased from bursa to stronger and relatively uniform staining in the SR, staining for Antr and CG1656 was already at maximal levels in the bursa and showed no further increase in the SR.

Several not-mutually-exclusive mechanisms could explain why different SFPs showed different spatiotemporal characteristics in their association with sperm. First, it could be that some LTR-SFPs catalyze each other’s binding, and thus that some need to bind earlier than others. The earliest-binding LTR-SFPs may facilitate some SP binding, but full binding requires the full complement of LTR-SFPs on sperm. At least one LTR-SFP (CG9997) is post-translationally modified (cleaved) within the female. The role of this cleavage is unknown, but one could imagine that modifications of this sort could also affect a protein’s binding to sperm, or its ability to catalyze another protein’s sperm binding.

Second, recently Wainwright et al. [54] showed that SP (at least) is transferred to females on large, neutral lipid-containing “microcarriers” that dissemble after entering the female reproductive tract, releasing their contents. It may be that the slow appearance of SP on sperm reflects its release by disassembly of these microcarriers; the timing that we observe is consistent with the timing of microcarrier dissociation reported by Wainright et al. [54]. A similar explanation could underlie the differences in sperm-binding kinetics of the LTR-SFPs, but it is not yet known whether they are transferred on microcarriers.

Third, recent results [36] show that as sperm transit through and remain in the FRT, female-derived proteins become associated with them. High enrichment of these female-derived sperm-associated proteins in energy metabolism pathways suggest that females contribute proteins that facilitate energy production and/or support sperm viability in storage, long term. It is also possible that some of these female proteins facilitate the binding of particular SFPs to sperm. Future studies are needed to determine if the association of individual SFPs with sperm is mediated or facilitated by any of these female-derived sperm-associated proteins, as well as the relative contributions of the two other mechanisms noted just above.

### Partial or greater loss of spermathecal secretory cells or parovaria does not affect the initial association of SFPs with sperm, but affects the release of SP from sperm

When we disabled SSCs by driving expression of Rh1^G69D^ [43, 44], or obtained full or partial loss of parovaria and SSCs with *Hr39* mutants [37], we did not observe detectable differences in the initial amount (or distribution) of SFP-sperm association relative to controls, indicating that secretions from SSCs and/or parovaria do not play a role, or at least a unique role, in facilitating the initial binding of SFPs to sperm. However, *Hr39* mutants differed from controls in the rate of release of SP from sperm; at 4d ASM, we observed higher retention of SP on sperm stored in *Hr39* mutant females relative to levels seen in controls. The impaired release of SP from sperm that we observed in *Hr39* females is expected to impair the rate by which they release sperm from storage, as SP activity is needed for this phenomenon [51]. Consistent with this expectation, we observed that *Hr39* mutant females from all five mutant lines showed significantly higher sperm counts in their SR at 8 days ASM, verifying that they had poor release of stored sperm relative to control females. Our results may provide a mechanism for the observations in two previous studies [40, 41] that secretions of the SSCs, or SSCs and parovaria, are necessary for stored sperm to be efficiently used for fertilization.

The release of SP’s C-terminal active region from sperm occurs by proteolysis [18], but the source of the protease that accomplishes this has been a mystery. Our results suggest that this protease may be derived from the female, and specifically from her SSCs or parovaria (or that its expression is regulated by the *Hr39* transcription factor). That the female would provide the protease to release SP to sperm makes sense physiologically, in that SFPs (other than SP) do not persist in the female for more than 1 day post-mating, making it likely that a male-derived protease that could cleave SP would not remain in the female long enough to regulate SP cleavage (unless the protease is a sperm-protein). It also raises interesting evolutionary implications that the female would provide the activity that permits the active portion of SP to be released and function.

## Conclusion

Our findings highlight that molecular contributions from both males and females are needed to facilitate association and/or dissociation of SFPs/SP to sperm and encourage future studies to identify the female candidates that mediate these molecular interactions between sexes.

## Methods

### Fly strains and crossing scheme

Flies used for ejaculate collections were derived from a cross between *UAS-dTrpAI* [55] and *UAS-mCD8-Gfp*; *fru*-GAL4(B)/*MKRS* [56]. The flies were a generous gift from the Baker lab (Janelia). *Fru*-GAL4>*UAS-dTrpAI* males expel ejaculate after exposure to heat (29°C) as a result of activation of the temperature-sensitive cation channel, dTrpA1. All stocks not otherwise indicated were obtained from the BDSC. To disrupt/abolish the secretory units of the female reproductive tract, *UAS-Rh1*^*G69D*^ flies (a generous gift from Dr. H.D. Ryoo [43]) were crossed to *Send1-GAL4; Gla/CyO* (specific to spermathecae; kind gift of Dr. M. Siegal) flies to induce tissue-specific generation of ER stress, and the ablation of secretory units (paraovaria and SSCs lining the spermathecal cap). We also used five publicly available *Hr39* mutant lines, y[1] w[*]; Mi{y[+mDint2]=MIC}Hr39[MI06174] (BL38620 [47;), w[*]; P{w[+mGS]=GSV1}Hr39[C277] (BL 43358 [48])*, y[1] w[67c23]; P{y[+mDint2] w[+mC]=EPgy2}Hr39[EY04579]* (BL20152 [49]*)*, *y[1] w[67c23]; Hr39[C105]* (BL64285 [46]) and *y[1] w[67c23]; P{w[+mW.hs]=GawB}Hr39[c739] P{w[+mC]=UAS-mCD8::GFP.L}LL5* (BL64305 [50])*.* Because balancer-sib controls (genetically matched controls) were either unavailable or sub-viable for 4/5 of the *Hr39* mutant lines, we used *Canton S* (CS) females as relative controls. *ProtB-eGFP* males with Protamine B-eGFP-tagged sperm heads were kindly gifted by the Pitnik lab [52]. All flies were reared under a 12:12h light-dark cycle at 22±1°C on standard yeast-glucose medium. Mating experiments were carried out by single-pair mating 3–5-day-old unmated control males to 3–5-day-old unmated females of the genotypes indicated in the text.

### Immunofluorescence

Immunostaining was performed to detect SP and LTR-SFPs binding to sperm as in [16, 17]. To obtain sperm isolated before mating: we heat-shocked *Fru*-GAL4>*UAS-dTrpAI* males at 29°C for 4 min to obtain the ejaculate. The expelled ejaculate was then pulled off from male’s external genitalia and collected in a drop of 1× PBS on the surface of poly-L-lysine-coated slides (Sigma). The mass of ejaculate was teased apart to separate sperm, which later adhere to the surface of the slides for further processing. Sperm isolated from male ejaculate were processed either immediately after exudation (Fig. 1I, sample A) or were incubated in 1× PBS for 2h after exudation from males (Fig. 1I, sample G). To obtain sperm after mating (in female bursa and seminal receptacle): *Canton S* (CS) females mated to CS males were used to collect sperm from bursa (35 min after the start of mating, ASM; Fig. 1I, sample C) and seminal receptacle (2 h ASM; Fig. 1I, sample E). For additional experiments, *Send1>Rh1*^*G69D*^, *Send1>CyO*, *Hr39 mutant* females and *balancer-sib* (or *CS*, where necessary) controls were mated to CS males.

Sperm were isolated from seminal receptacle of these mated females at 2 h and/or 4 days ASM. All the samples were processed according to the protocol of Ravi Ram and Wolfner [4] with minor modifications. Samples were blocked with 5% bovine serum albumin, BSA in 1× PBS for 30 min. Subsequently, samples were incubated overnight in rabbit anti-SP(1:200), CG1656(1:100), CG1652(1:50), CG9997(1:50) [4, 22, 57] in 0.1% BSA at 4°C. Samples were then washed in 1× PBS and incubated at room temperature for 2 h in mouse anti-rabbit IgG coupled to Alexa fluor 488 (green; Invitrogen) at a concentration of 1:300 in 1× PBS at room temperature in the dark. Samples were then washed in PBS, incubated in 0.01% DAPI for 3 min at room temperature in the dark, rewashed and mounted using antifade (CitiFluor mountant solution; EMS). Fluorescence was visualized under an Echo-Revolve fluorescence microscope at a magnification of 20X. The intensity of anti-SFP immunofluorescence on sperm tails was quantified using ImageJ software (National Institute of Health, Bethesda, USA). The region of interest (ROI) was selected as the rectangular area of 784 AU flanking the individual sperm flagellum (sperm tail) right underneath the DAPI (blue) and SP (green) stained cyan sperm head to keep the consistency in between groups and across the samples. The corrected fluorescence (CF) of the samples were calculated by deducting the integrated density values of background with those of samples. The difference in the fluorescence intensity of anti-SFP on sperm tails between ejaculate, bursa, SR samples, and SR samples in different *Hr39* lines used at different time points (2 h and 4 days ASM) were statistically analyzed using one-way analysis of variance (ANOVA), followed by Tukey’s multiple comparison tests. The difference in the fluorescence intensity of anti-SP on sperm tails between *Send1>Rh1*^*G69D*^ and *Send1>CyO* females or BL64285 and their balancer-sib controls at different time points (2 h and 4 days ASM) were analyzed statistically using unpaired *t*-tests. A minimum of three independent immunostaining batches, each with a minimum sample size of 8–15, were analyzed for each group.

In the tests of SP binding to sperm from ejaculate or in storage in control females, the genetic background and/or heat treatment of males we used did not affect the levels of SP associated with sperm. For ejaculate extrusion, we needed to use *Fru*-GAL4>*UAS-dTrpA1* males exposed to 29°C for 4 min. While it would have been ideal to use sperm from the same males, treated the same way, to examine SP binding to sperm from females’ bursa (35 min ASM) or SR (2 h ASM), heat-treated *Fru-GAL4*>*UAS-dTrpA1* males could not be used for matings with females, since the males were drowsy, as well as often stuck together with ejaculate. Therefore, we used *CS* males for matings to obtain and examine sperm isolated from the bursa and SR. To verify that the genotype and/or heat treatment did not affect the binding that we saw at 2 h ASM, we isolated sperm from SR of *CS* females mated to the following: non-heat-shocked (NHS) *Fru*-GAL4>*UAS-dTrpA1* males (Additional file 1: Fig. S1. A) that had the same genetic background as the males from which we obtained ejaculate; heat-shocked (29°C for 4 min; HS) *CS* males (Additional file 1: Fig. S1. B) that were of the same genotype we used in Fig. 1E–E’, but were exposed to the heat shock conditions used for ejaculate extrusion, and NHS *CS* males (Additional file 1: Fig. S1. C) that were previously used in our experiments (Fig. 1E–E’). We performed immunofluorescence to probe the levels of SP associated with sperm in these samples; the samples were processed, imaged, and quantified in exactly the same way and with similar dilutions for anti-SP as described above. As we were unable to tease out individual sperm in the samples, we measured fluorescence in the ROI of a rectangular area of 880 AU in the sperm aggregates. Two independent immunostaining batches were analyzed for each group. The difference in the fluorescence intensity of anti-SP on sperm aggregates between three groups were statistically analyzed using one-way analysis of variance (ANOVA), followed by Tukey’s multiple comparison tests. We observed no striking difference in the levels of SP associated with stored sperm from NHS *Fru-GAL4*>*UAS-dTrpA1* or HS *CS* males, relative to the levels seen for sperm from NHS *CS* male (Additional file 1: Fig. S1. D), indicating no apparent effect of genotype or heat treatment on levels of SP associated with sperm stored in SR at 2 h ASM.

### Efficacy of ablation of SSCs

The reproductive tracts from *Send1>Rh1*^*G69D*^ and *Hr39* mutant females were dissected and analyzed to detect the presence of ablated SSCs, if any. Whole female reproductive tracts were dissected in 1× PBS on a slide. The tissues were fixed with 4% paraformaldehyde (PFA) for 15 min. Samples were then washed in 1× PBS, incubated in 0.01% DAPI for 3 min at room temperature in the dark, rewashed, and mounted using antifade (CitiFluor mountant solution; EMS). Images were captured through an Echo-Revolve fluorescence microscope at a magnification of 20X. A minimum of two independent batches, with a minimum sample size of five per batch, were analyzed for each group.

Ablation of spermathecal secretory cells (SSCs) in the female reproductive tract varied in degree and penetrance of the phenotype across the lines tested. We ablated the SSCs that line the spermathecal cap by driving the expression of misfolded protein Rh1^G69D^ in these cells [43, 44]. Expression of Rh1^G69D^ induces excessive ER stress in the targeted tissues, disrupting protein synthesis/secretion by the cells. We used *Send1-Gal4* to drive the expression of Rh1^G69D^ in the female reproductive tract. *Send1>Rh1*^*G69D*^ females had ablated SSCs, but the penetrance of the phenotype was not complete: some of the mosaic females (3/5) had only one of the two spermathecae lacking SSCs and the other spermatheca still had numerous SSCs (Fig. 4C, D). Control *Send1>CyO* females had normal numbers of SSCs around their spermathecal caps (Fig. 4A, B; DAPI stained).

As an alternative way to ablate SSCs, we used mutants of the nuclear hormone receptor Hr39 [37, 40], which is needed for the formation of secretory units in the female reproductive tract. Loss of expression of Hr39 affects the development of SSCs and parovaria. We focused on *Hr39* mutants that exhibit more stringent phenotypes for SSC ablation. We tested five different *Hr39* mutants—BL38620 (*Hr39[MI06174]*), BL43358 (*Hr39[C277]*), BL20152 (*Hr39[EY04579]*), BL64285 (*Hr39[C105]*), and BL64305 (*Hr39[c739]*) to determine the extent of loss of SSCs in mutant females from each stock relative to control females. Ablated SSCs were observed around the spermathecal caps in the reproductive tracts of all the five *Hr39* mutant lines, but again, the penetrance of phenotype varied in SSC numbers (or size). As expected, CS females had large, regular SSCs (approximately 60–80 cells) lining both spermathecal caps (Additional file 4: Fig. S4. A and B; DAPI stained). *Hr39* mutant BL 38620 females gave us the same pattern of SSC ablation as was observed in *Send1>Rh1*^*G69D*^ females where some females lacked SSCs in both spermathecae (3/5), while in the other females (2/5) only one of the two spermathecae lacked SSCs (Additional file 4: Fig. S4. C and D; DAPI stained). *Hr39* mutant females in stocks BL43358 (Additional file 4: Fig. S4. E and F; DAPI stained) and BL 64285 (Additional file 4: Fig. S4. I and J; DAPI stained) had completely ablated SSCs in some females (4/5) and extremely reduced numbers and sizes of the SSCs lining the spermathecal cap, in others (1/5). *Hr39* mutant females from the other two stocks, BL 20152 (Additional file 4: Fig. S4. G and H; DAPI stained) and BL64305 (Additional file 4: Fig. S4. K and L; DAPI stained) had almost complete ablation of SSCs from both the spermathecae, except that 3–4 SSCs could still be seen in some (1/5) of the females.

### Sample preparation and western blotting

To determine the binding of SP and LTR-SFPs to sperm and persistence of sperm-bound SP long term, sperm stored (SS) in the seminal receptacle of females (of the indicated genotype) mated to control males were dissected. The dissected tissues (SS, *n*=30) were suspended in 5μl of homogenization buffer (5% 1M Tris; pH 6.8, 2% 0.5M EDTA) and processed further according to the protocol of Ravi Ram and Wolfner [4]. Proteins from stored sperm were then resolved on 12% polyacrylamide SDS gel and processed further for western blotting. Affinity purified rabbit antibodies against SP (1:2000), CG1656 (1:1000), CG1652 (1:500), antares (1:500), CG9997 (1:1000), CG17575 (1:1000), seminase (1:1000) [4, 16, 22], and mouse antibody against tubulin (as a loading control; 1:3500) were used as primary antibodies. HRP conjugated secondary anti-rabbit and anti-mouse antibodies (Jackson Research) were used for detection of SFPs at a concentration of 1:2000. The levels of SP were normalized with tubulin of respective lanes using Quantity One software. The experiments were performed in triplicate and gave comparable results, but due to variability in the background levels, data from individual blots were used for statistical analyses. The difference in the signal intensity of SP levels on western blots between BL64285 and their balancer-sib controls (Fig. 4B, C) and different *Hr39* lines and their relative *CS* controls, at 2 h (Additional file 5: Fig. S5B) and 4 days ASM (Additional file 7: Fig. S7B) were analyzed statistically using unpaired *t*-tests, and one-way analysis of variance (ANOVA), followed by Tukey’s multiple comparison tests, respectively.

### Sperm release from sperm storage organs in females

To study the sperm utilization and release, *Hr39* mutant females mated to *ProtB-eGFP* (control) males were frozen at 8 days ASM for sperm counts. Subsequently, seminal receptacles of mated females were dissected and eGFP sperm were counted (at a total magnification of 20X, with FITC filter on an Echo-Revolve microscope). Mature sperm in the seminal receptacles of mated females were counted twice and groups were blinded to ensure reproducibility and avoid bias [58]. The percent repeatability was 88–92% across the samples. Assays were repeated twice, with two technical replicates. Differences in the sperm counts between groups were analyzed statistically through one-way ANOVA followed by Tukey’s multiple comparison tests. Each group contained a minimum sample size of 15–25.

## Supplementary Information

**Additional file 1: Figure S1.** No striking change in the levels of SP associated with sperm stored in SR of CS females mated to males with different genotypes and exposure to heat conditions. Sperm isolated from the seminal receptacle (SR) of wildtype (CS) females, frozen at 2 h ASM after mating with (A) non-heat-shocked (NHS) *Fru*-GAL4>*UAS-dTrpA1* males, (B) CS males heat-shocked (HS) at 29°C for 4 min before the start of mating, and (C) CS males, NHS. Sperm heads were stained with DAPI (blue) and anti-SP staining was visualized with Alexa fluor 488, staining the sperm tail (green) and sperm head (cyan; overlapping blue/green); Bar = 20μm. The insets show the negative controls for their respective panels. Sperm samples in negative controls were incubated with only secondary antibody (anti-rabbit, Alexa fluor 488), with no primary antibody (anti-SP) incubation. (D) shows corrected fluorescence (CF) intensity of SP on sperm stored in SR of females, received from males with difference in genetic background and exposure to heat conditions. Since we were not able to tease-out individual sperm in these samples, we measured the signal at 5 randomly-selected positions per sperm aggregate, using the same size and shape of ROI for each measurement. Measurements are plotted in the graph, with bars showing Mean±SE (AU stands for arbitrary units); p=0.7781, ns=not significant; degree of freedom, F(2,12)= 0.2562.

**Additional file 2: Figure S2.** CG17575 and Seminase do not associate with sperm at any stage from male ejaculate to female storage. Pre-mating ejaculate samples were collected from *Fru>dTRPA1* males exposed to high temperatures, as described in Methods. Post-mating sperm samples were isolated from mated females. Wild type (*CS*) females were mated to a wildtype (*CS*) male and frozen at 35 min (sperm in bursa) and 2 h (sperm stored in seminal receptacle) ASM. Sperm heads were stained with DAPI (blue) and seminase and CG17575 were visualized with Alexa fluor 488 (green). Sperm isolated from male ejaculate (A and D) probed for seminase and CG17575, respectively. Sperm isolated from female bursa at 35 min ASM (B and E) probed for seminase and CG17575, respectively. Sperm isolated from female seminal receptacle at 2 h ASM (C and F) probed for seminase and CG17575, respectively. (n=10; Bar = 20μm).

**Additional file 3: Figure S3.** Anti-SP staining on sperm dissected from *Send1> Rh1* females (with ablated SSCs) do not show any difference in SP levels when compared to their levels in matched-control females at 2 h or 4 days ASM. Sperm samples isolated from the seminal receptacle of *Send1>CyO* (Control) females (A-A’) and *Send1>Rh1* (experimental) females (B-B’) mated with CS males and frozen at 2 h ASM. Sperm samples isolated from the seminal receptacle of *Send1>CyO* (Control) females (C-C’) and *Send1>Rh1* (experimental) females (D-D’) mated with CS males and frozen at 4 days ASM. Sperm heads were stained with DAPI (blue) and anti-SP staining was visualized with Alexa fluor 488, staining the sperm tail (green) and sperm head (cyan; overlapping blue/green). Insets show the respective negative controls for each panel, with only secondary antibody (anti-rabbit, Alexa fluor 488) and no primary antibody (anti-SP) incubation. Panels A’, B’, C’, D’ have an added transmitted light filter to show the outlines of sperm tails in the regions where SP was undetected (e.g, panel C and D), n=11-7; Bar = 20μm (E) Corrected fluorescence (CF)intensity of SP bound to stored sperm in *Send1>CyO* (control) and *Send1>Rh1* females at 2 h ASM; p=0.2818; ns= non significant; Error bars show Mean±SE AU (AU stands for arbitrary units); F(10,10)=2.023. (F) Corrected fluorescence intensity of SP bound to stored sperm in *Send1>CyO* (control) and *Send1>Rh1* females at 4 days ASM; Error bars show Mean±SE AU.

**Additional file 4: Figure S4.** *Hr39* mutant females show either completely ablated or extremely reduced SSC numbers. Control or mutant females were mated with *ProtB-eGFP* males (eGFP-tagged sperm; green). SSCs, marked with DAPI stained nuclei (cells enclosed in white dotted circle) lining the spermathecal cap (red dotted circle). (A-B) Control (CS) females show normal bunch of SSCs around both the spermathecal caps. Completely ablated SSCs or SSCs reduced in cell size or number were observed in the *Hr39* mutant lines (C-D) BL38620, (E-F) BL 43358, (G-H) BL20152, (I-J) BL 64285, (K-L) BL 64305. n=5; Bar = 20μm.

**Additional file 5: Figure S5.** Initial SP binding with sperm dissected from *Hr39* mutant females (with ablated SSCs) do not show any difference in SP levels when compared to their levels in CS females at 2 h ASM. (A) Western blot probed for SP at 2 h ASM. Lanes# 1: Fv, reproductive tract (RT) of 4 virgin females (negative control), 2: MAG, 1 pair of male accessory glands (positive control), 3: CS, sperm dissected from SR of 30 control (*CS)* females mated to wild type (CS) males, 4: 38620, sperm dissected from SR of 30 *Hr39* mutant (BL38620) females mated to wild type (CS) males, 5: 43358, sperm dissected from SR of 30 *Hr39* mutant (BL43358) females mated to wild type (CS) males, 6: 20152, sperm dissected from SR of 30 *Hr39* mutant (BL20152) females mated to wild type (CS) males, 7: 64285, sperm dissected from SR of 30 *Hr39* mutant (BL64285) females mated to wild type (CS) males, 8: 64305, sperm dissected from SR of 30 *Hr39* mutant females mated to wild type (CS) males. Tubulin (Tub) served as the loading control. (B) Graphical representation of the normalized levels of sperm-bound SP in *Hr39* mutant (red bars) females from all the five stocks relative to CS females (blue bar & blue dotted line) at 2 h ASM, as seen on one of three replicate Western blots; the other two blots showed similar results (ns=non significant) Sperm samples isolated from the seminal receptacle of CS (control) females (C), and other four *Hr39* mutant females, BL38620 (D), BL43358 (E), BL20152 (F), BL64305 (G). The females were mated with CS males and frozen at 2 h ASM. Sperm heads were stained with DAPI (blue) and anti-SP staining was visualized with Alexa fluor 488, staining the sperm tail (green) and sperm head (cyan; overlapping blue/green). Insets show the respective negative controls for each panel, with only secondary antibody (anti-rabbit, Alexa fluor 488) and no primary antibody (anti-SP) incubation, n=11; Bar = 20μm (H) Graphical representation of corrected fluorescence intensity of SP bound to stored sperm in CS females (control) and other four *Hr39* females, BL38620, BL43358, BL20152 and BL64305 at 2 h ASM; p=0.449; ns= nonsignificant; F(4,50)=0.9468. Error bars show Mean±SE AU.

**Additional file 6: Figure S6.** *Hr39* mutant females have normal binding of LTR-SFPs to sperm but excessively retain sperm in storage. (A) Western blot probed for LTR-SFPs, at 2 h ASM. Lanes# 1: Fv, reproductive tract (RT) of 4 virgin females (negative control), 2: MAG, 1 pair of male accessory glands (positive control), 3: CS, sperm dissected from SR of 30 control (CS) females mated to wild type (CS) males, 4: 38620, sperm dissected from SR of 30 *Hr39* mutant (BL38620) females mated to wild type (CS) males, 5: 43358, sperm dissected from SR of 30 *Hr39* mutant (BL43358) females mated to wild type (CS) males, 6: 20152, sperm dissected from SR of 30 *Hr39* mutant (BL20152) females mated to wild type (CS) males, 7: 64285, sperm dissected from SR of 30 *Hr39* mutant (BL64285) females mated to wild type (CS) males, 8: 64305, sperm dissected from SR of 30 *Hr39* mutant (BL64305) females mated to wild type (CS) males. Lanes were probed for LTR- SFPs , CG1656, CG1652, Antares and CG9997 as described in the text. (B) Graphical representation of sperm counts in SRs of CS and *Hr39* females from all the five mutant stocks, mated to control *ProtB-eGFP* males (with eGFP tagged sperm; green; error bars show mean±SE; p**=<0.01; n=15-20) and frozen at 8 days ASM.

**Additional file 7: Figure S7.** SP levels on sperm after normalization of Western blot and anti-SP staining on sperm dissected from *Hr39* mutant females (with ablated SSCs and parovaria) show higher levels of SP levels when compared to their levels in sib-control or CS females at 4 days ASM. (A) Western blot probed for SP at 4 days ASM. Lanes# 1: Fv, reproductive tract (RT) of 4 unmated females (negative control), 2: MAG, 1 pair of male accessory glands (positive control), 3: CS, sperm dissected from SR of 30 control (*CS)* females mated to wild type (CS) males, 4: 38620, sperm dissected from SR of 30 *Hr39* mutant (BL38620) females mated to wild type (CS) males, 5: 43358, sperm dissected from SR of 30 *Hr39* mutant (BL43358) females mated to wild type (CS) males, 6: 20152, sperm dissected from SR of 30 *Hr39* mutant (BL20152) females mated to wild type (CS) males, 7: 64285, sperm dissected from SR of 30 *Hr39* mutant (BL64285) females mated to wild type (CS) males, 8: 64305, sperm dissected from SR of 30 *Hr39* mutant (BL64305) females mated to wild type (CS) males. Tubulin (Tub) served as the loading control. (B) Graphical representation of the normalized levels of sperm bound SP in *Hr39* mutant (red bars) females from all the five stocks relative to CS females (blue bar & dotted line) at 4 days ASM, as seen on one of three replicate Western blots; the other two blots showed similar results. Sperm samples isolated from the seminal receptacle of (C) CS (control) females and the other four *Hr39* mutant females, (D) BL38620, (E) BL43358, (F) BL20152, (G) BL64305. The females were mated with CS males and frozen at 4 days ASM. Sperm heads were stained with DAPI (blue) and anti-SP staining was visualized with Alexa fluor 488, staining the sperm tail (green) and sperm head (cyan; overlapping blue/green). The insets show the respective negative controls for their panels. The larger panels have transmitted light filter added to show the outline of sperm tail in the regions where SP was undetected (e.g, panel C); n=10; Bar = 20μm. (H) Graphical representation of corrected fluorescence (CF) intensity of SP bound to stored sperm in CS females (control) and other four *Hr39* females, BL38620, BL43358, BL20152 and BL64305 at 4 days ASM; p***<0.001; n=10; F(4,45)=23.16. Error bars show Mean±SE AU.

**Additional file 8: Figure S8.** Levels of SP transferred to the FRT and bound to sperm stored in SR of balancer-sib control for BL64285 and CS females, do not show any significant difference at 2 h ASM, suggesting no evident background effect. Western blot probed for SP Lanes# 1: Fv, reproductive tract (RT) of 4 unmated females (negative control), 2: MAG, 1 pair of male accessory glands (positive control), 3: Ctl, reproductive tract (RT) of 4 balancer-sib (BL64285 {*Hr39[C105]*/CyO) control females mated to wild type (CS) males at 35 min ASM, 4: CS, RT of 4 CS females mated to wild type (CS) males at 35 min ASM, 5: Ctl sperm dissected from SR of 30 balancer-sib control females mated to wild type (CS) males, at 2 h ASM, 6: CS, sperm dissected from SR of 30 CS females mated to wild type (CS) males at 2 h ASM. Tubulin (Tub) served as the loading control.

**Additional file 9.** Uncropped blots.

## Data Availability

All data generated or analyzed during this study are included in this published article and its additional files.

## Abbreviations

SFPs: Seminal fluid proteins
SP: Sex Peptide
LTR-SFPs: Long-term response SFPs
FRT: Female reproductive tract
SSCs: Spermathecal secretory cells
ST: Spermathecae
SR: Seminal receptacle
SPR: Sex Peptide Receptor
ASM: After the start of mating
AU: Arbitrary units
F: Degree of freedom
SE: Standard error
PBS: Phosphate buffer saline
Antr: Antares
ns: Nonsignificant
ER: Endoplasmic reticulum
NR: Nuclear receptor
*Hr39*: Hormone receptor-like in 39
Rh: Rhodopsin
Cy: Curly
CS: Canton S
-sib: Sibling
ProtB-eGFP: Protamine B-enhanced GFP
CF: Corrected fluorescence
Esp: Epidermal stripes and patches
BSA: Bovine serum albumin
ROI: Region of interest
ANOVA: Analysis of variance
PFA: Paraformaldehyde
SS: Sperm stored
DAPI: 4′,6-Diamidino-2-phenylindole
EDTA: Ethylene diamine tetra-acetic acid
SDS: Sodium do decyl sulfate
HRP: Horseradish peroxidase
FITC: Fluorescein isothiocyanate
FRU: Fruitless
NHS: Non-heat shocked
