# Supplementary figures and images for: Female factors modulate Sex Peptide’s association with sperm in Drosophila melanogaster
Source: BMC Biol. 2022 Dec 14;20:279. doi: 10.1186/s12915-022-01465-2 (PMC9749180; doi:10.1186/s12915-022-01465-2)

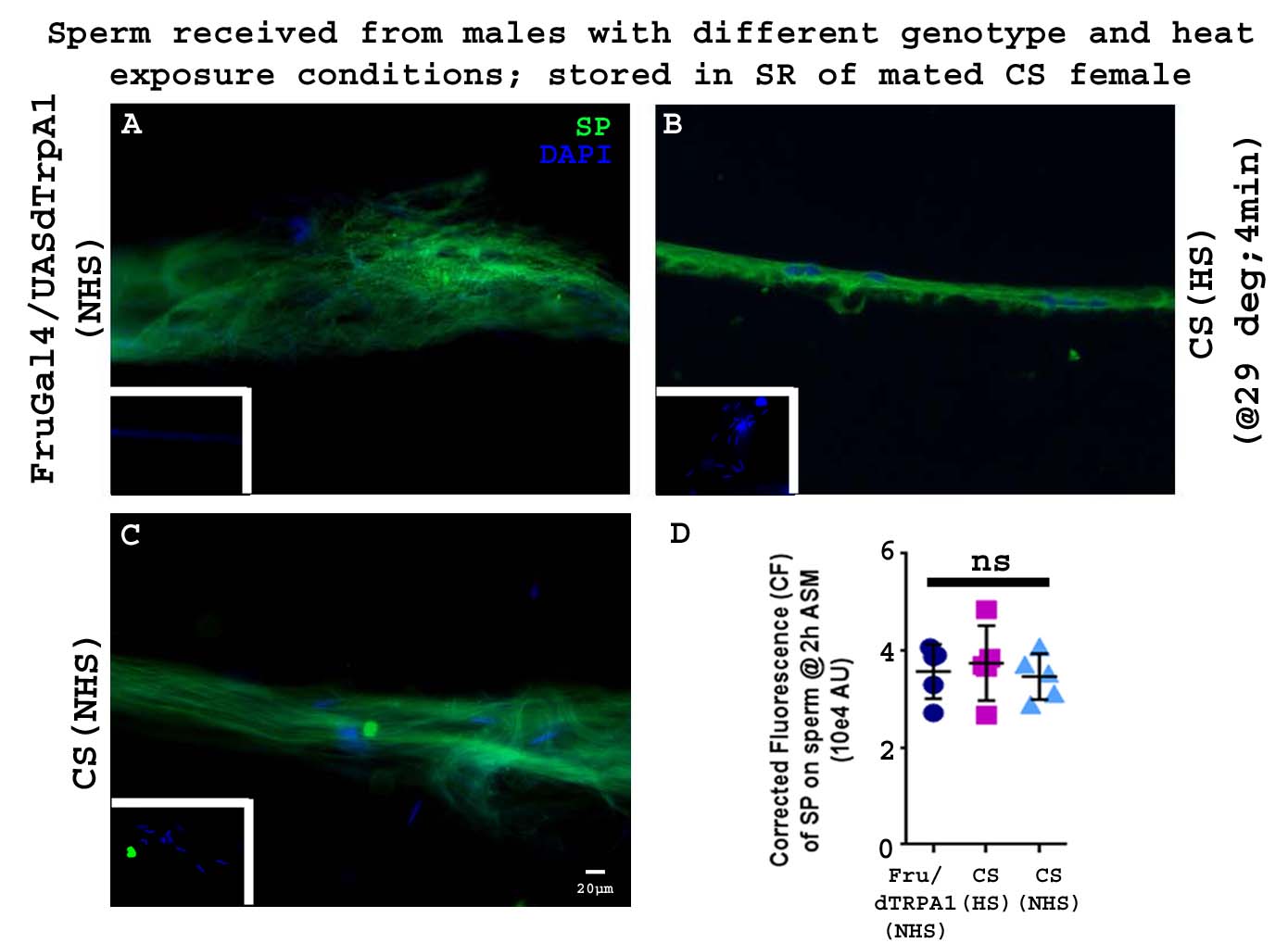

Supplement: Supplementary file 1 — Additional file 1: Figure S1. No striking change in the levels of SP associated with sperm stored in SR of CS females mated to males with different genotypes and exposure to heat conditions. Sperm isolated from the seminal receptacle (SR) of wildtype (CS) females, frozen at 2 h ASM after mating with (A) non-heat-shocked (NHS) Fru-GAL4>UAS-dTrpA1 males, (B) CS males heat-shocked (HS) at 29°C for 4 min before the start of mating, and (C) CS males, NHS. Sperm heads were stained with DAPI (blue) and anti-SP staining was visualized with Alexa fluor 488, staining the sperm tail (green) and sperm head (cyan; overlapping blue/green); Bar = 20μm. The insets show the negative controls for their respective panels. Sperm samples in negative controls were incubated with only secondary antibody (anti-rabbit, Alexa fluor 488), with no primary antibody (anti-SP) incubation. (D) shows corrected fluorescence (CF) intensity of SP on sperm stored in SR of females, received from males with difference in genetic background and exposure to heat conditions. Since we were not able to tease-out individual sperm in these samples, we measured the signal at 5 randomly-selected positions per sperm aggregate, using the same size and shape of ROI for each measurement. Measurements are plotted in the graph, with bars showing Mean±SE (AU stands for arbitrary units); p=0.7781, ns=not significant; degree of freedom, F(2,12)= 0.2562. [file 12915_2022_1465_MOESM1_ESM.jpg]

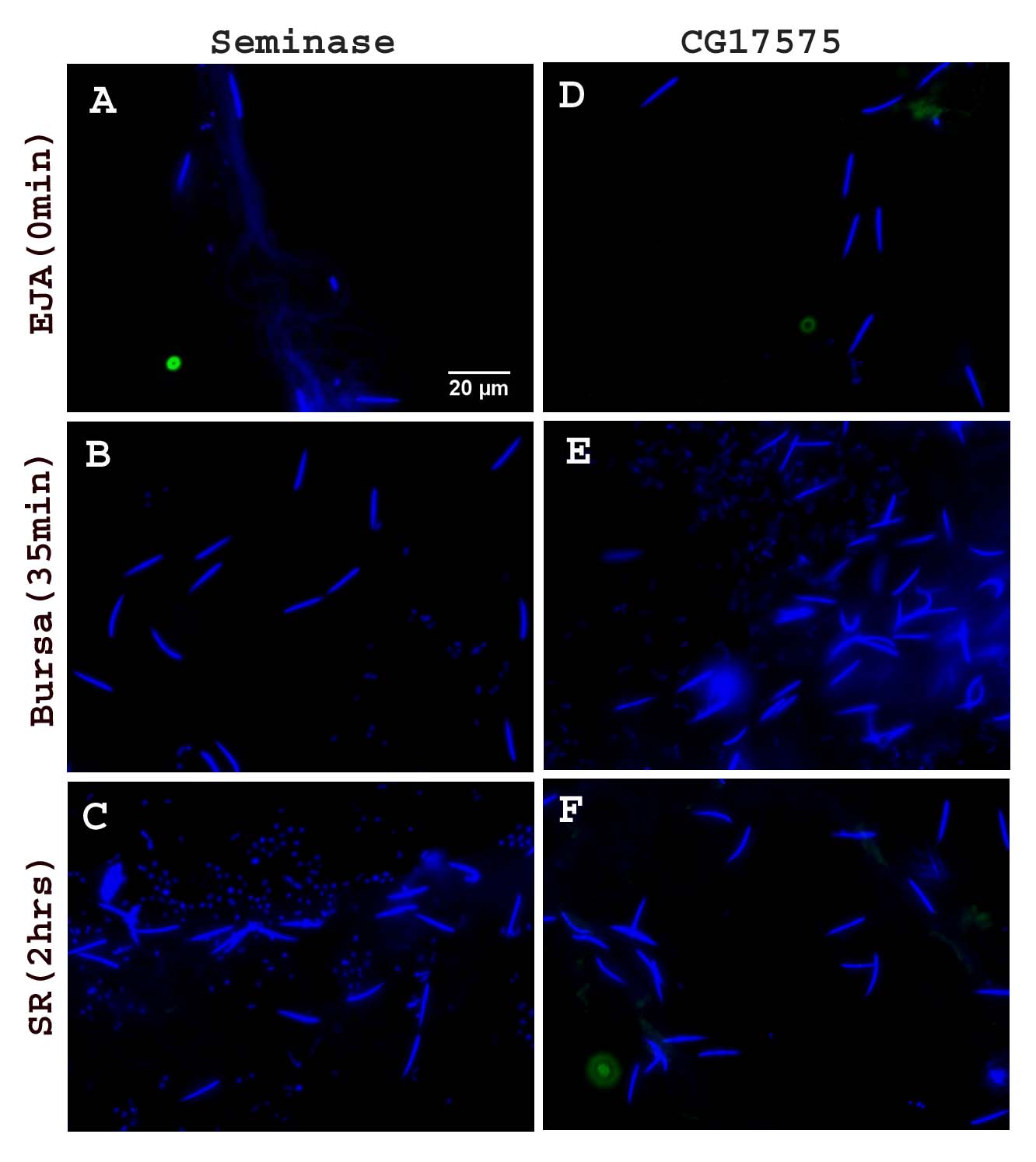

Supplement: Supplementary file 2 — Additional file 2: Figure S2. CG17575 and Seminase do not associate with sperm at any stage from male ejaculate to female storage. Pre-mating ejaculate samples were collected from Fru>dTRPA1 males exposed to high temperatures, as described in Methods. Post-mating sperm samples were isolated from mated females. Wild type (CS) females were mated to a wildtype (CS) male and frozen at 35 min (sperm in bursa) and 2 h (sperm stored in seminal receptacle) ASM. Sperm heads were stained with DAPI (blue) and seminase and CG17575 were visualized with Alexa fluor 488 (green). Sperm isolated from male ejaculate (A and D) probed for seminase and CG17575, respectively. Sperm isolated from female bursa at 35 min ASM (B and E) probed for seminase and CG17575, respectively. Sperm isolated from female seminal receptacle at 2 h ASM (C and F) probed for seminase and CG17575, respectively. (n=10; Bar = 20μm). [file 12915_2022_1465_MOESM2_ESM.jpg]

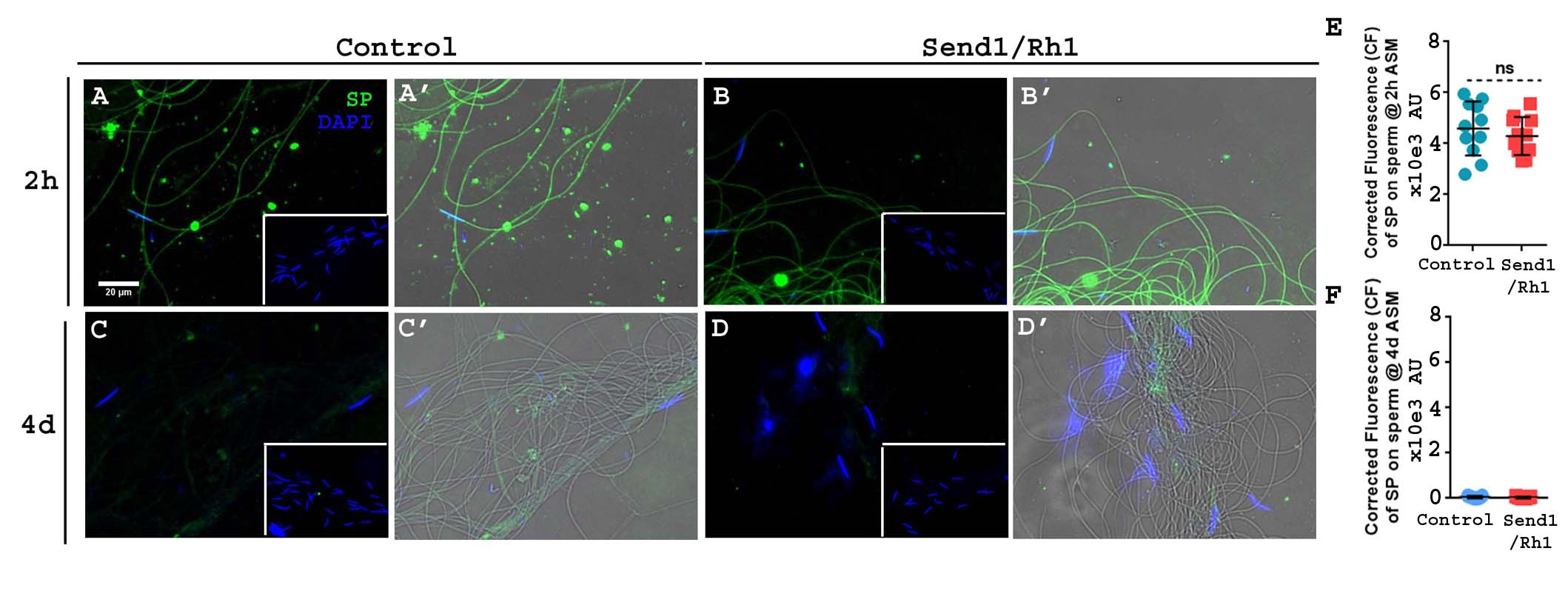

Supplement: Supplementary file 3 — Additional file 3: Figure S3. Anti-SP staining on sperm dissected from Send1> Rh1 females (with ablated SSCs) do not show any difference in SP levels when compared to their levels in matched-control females at 2 h or 4 days ASM. Sperm samples isolated from the seminal receptacle of Send1>CyO (Control) females (A-A’) and Send1>Rh1 (experimental) females (B-B’) mated with CS males and frozen at 2 h ASM. Sperm samples isolated from the seminal receptacle of Send1>CyO (Control) females (C-C’) and Send1>Rh1 (experimental) females (D-D’) mated with CS males and frozen at 4 days ASM. Sperm heads were stained with DAPI (blue) and anti-SP staining was visualized with Alexa fluor 488, staining the sperm tail (green) and sperm head (cyan; overlapping blue/green). Insets show the respective negative controls for each panel, with only secondary antibody (anti-rabbit, Alexa fluor 488) and no primary antibody (anti-SP) incubation. Panels A’, B’, C’, D’ have an added transmitted light filter to show the outlines of sperm tails in the regions where SP was undetected (e.g, panel C and D), n=11-7; Bar = 20μm (E) Corrected fluorescence (CF)intensity of SP bound to stored sperm in Send1>CyO (control) and Send1>Rh1 females at 2 h ASM; p=0.2818; ns= non significant; Error bars show Mean±SE AU (AU stands for arbitrary units); F(10,10)=2.023. (F) Corrected fluorescence intensity of SP bound to stored sperm in Send1>CyO (control) and Send1>Rh1 females at 4 days ASM; Error bars show Mean±SE AU. [file 12915_2022_1465_MOESM3_ESM.jpg]

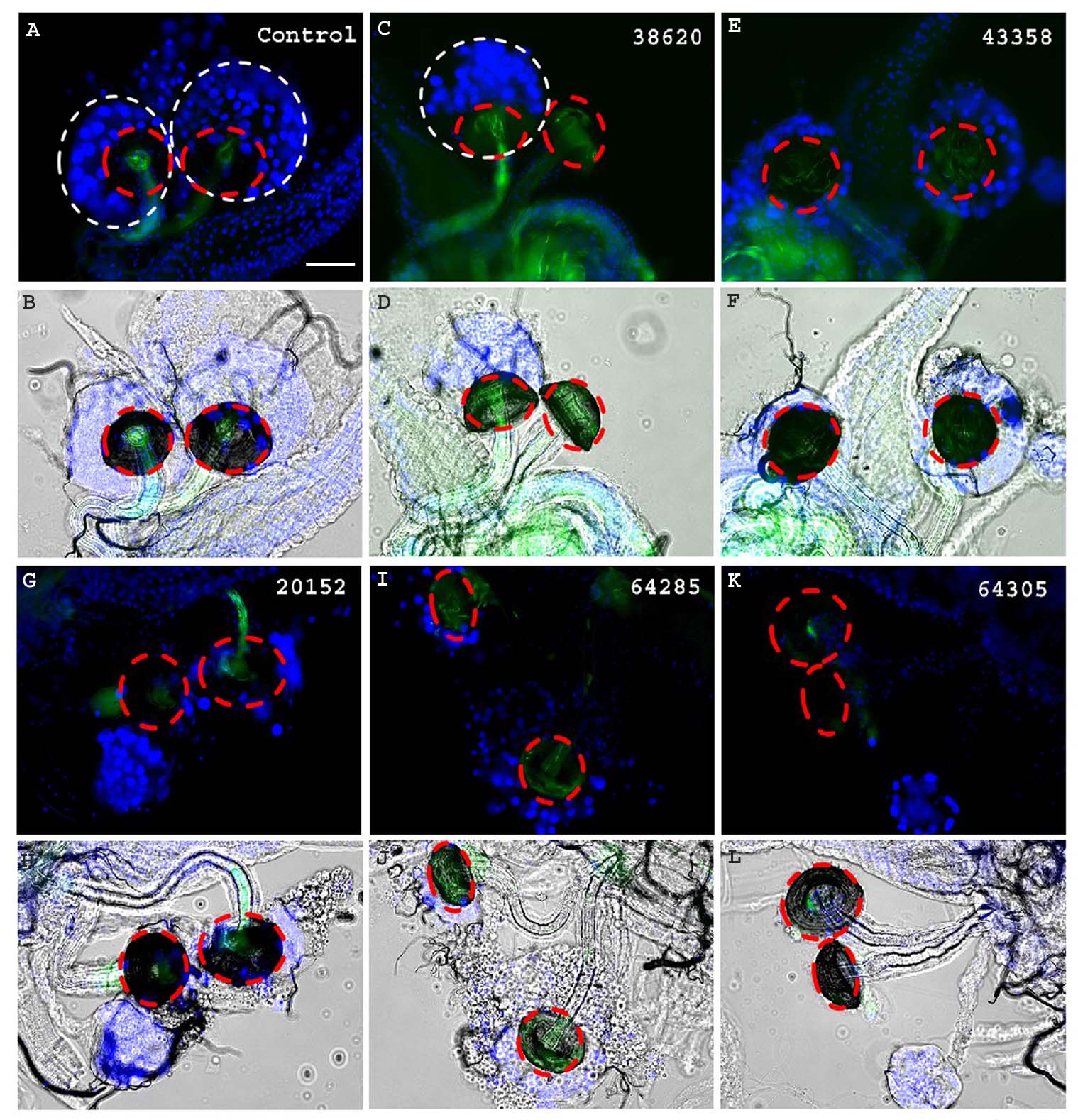

Supplement: Supplementary file 4 — Additional file 4: Figure S4. Hr39 mutant females show either completely ablated or extremely reduced SSC numbers. Control or mutant females were mated with ProtB-eGFP males (eGFP-tagged sperm; green). SSCs, marked with DAPI stained nuclei (cells enclosed in white dotted circle) lining the spermathecal cap (red dotted circle). (A-B) Control (CS) females show normal bunch of SSCs around both the spermathecal caps. Completely ablated SSCs or SSCs reduced in cell size or number were observed in the Hr39 mutant lines (C-D) BL38620, (E-F) BL 43358, (G-H) BL20152, (I-J) BL 64285, (K-L) BL 64305. n=5; Bar = 20μm. [file 12915_2022_1465_MOESM4_ESM.jpg]

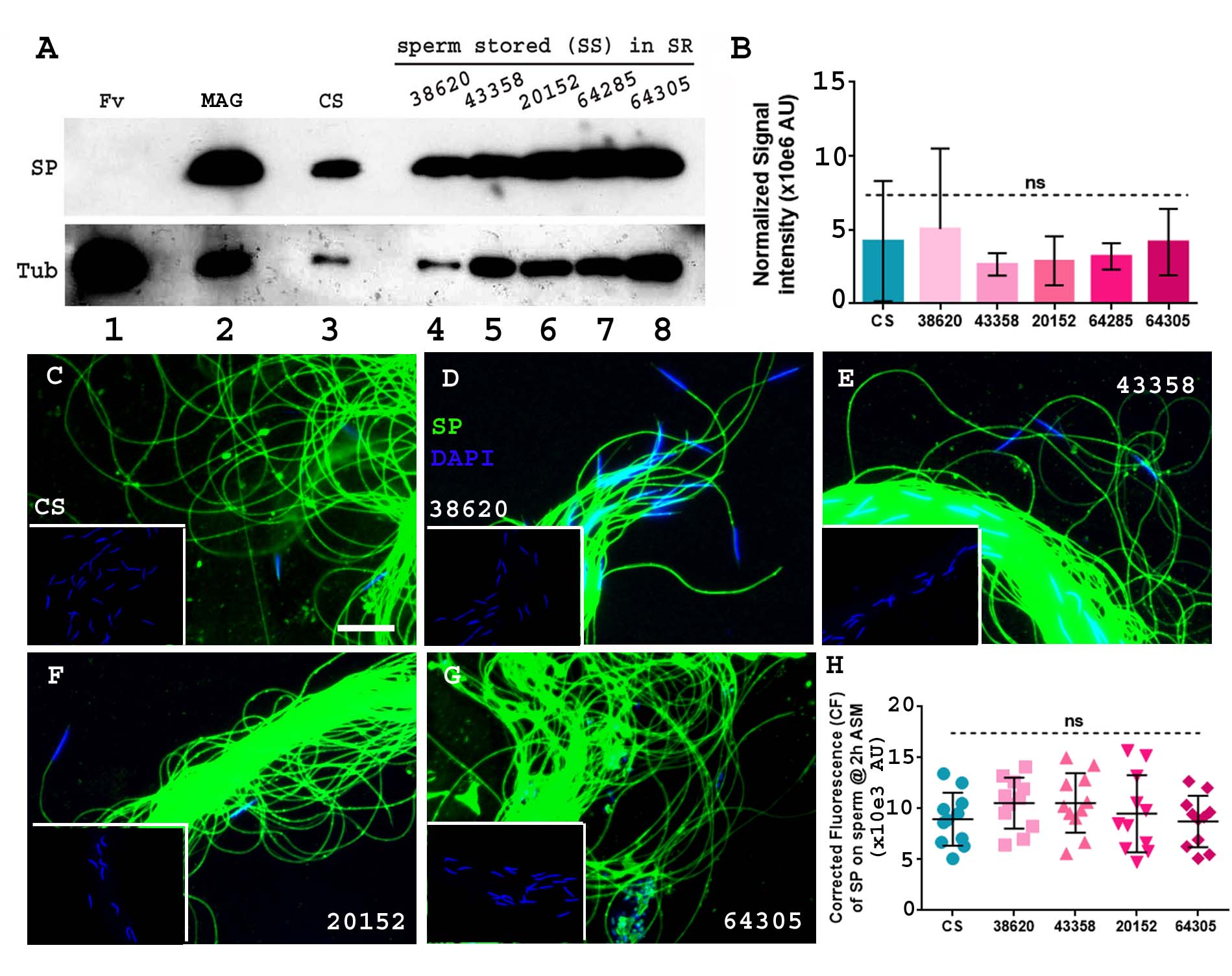

Supplement: Supplementary file 5 — Additional file 5: Figure S5. Initial SP binding with sperm dissected from Hr39 mutant females (with ablated SSCs) do not show any difference in SP levels when compared to their levels in CS females at 2 h ASM. (A) Western blot probed for SP at 2 h ASM. Lanes# 1: Fv, reproductive tract (RT) of 4 virgin females (negative control), 2: MAG, 1 pair of male accessory glands (positive control), 3: CS, sperm dissected from SR of 30 control (CS) females mated to wild type (CS) males, 4: 38620, sperm dissected from SR of 30 Hr39 mutant (BL38620) females mated to wild type (CS) males, 5: 43358, sperm dissected from SR of 30 Hr39 mutant (BL43358) females mated to wild type (CS) males, 6: 20152, sperm dissected from SR of 30 Hr39 mutant (BL20152) females mated to wild type (CS) males, 7: 64285, sperm dissected from SR of 30 Hr39 mutant (BL64285) females mated to wild type (CS) males, 8: 64305, sperm dissected from SR of 30 Hr39 mutant females mated to wild type (CS) males. Tubulin (Tub) served as the loading control. (B) Graphical representation of the normalized levels of sperm-bound SP in Hr39 mutant (red bars) females from all the five stocks relative to CS females (blue bar & blue dotted line) at 2 h ASM, as seen on one of three replicate Western blots; the other two blots showed similar results (ns=non significant) Sperm samples isolated from the seminal receptacle of CS (control) females (C), and other four Hr39 mutant females, BL38620 (D), BL43358 (E), BL20152 (F), BL64305 (G). The females were mated with CS males and frozen at 2 h ASM. Sperm heads were stained with DAPI (blue) and anti-SP staining was visualized with Alexa fluor 488, staining the sperm tail (green) and sperm head (cyan; overlapping blue/green). Insets show the respective negative controls for each panel, with only secondary antibody (anti-rabbit, Alexa fluor 488) and no primary antibody (anti-SP) incubation, n=11; Bar = 20μm (H) Graphical representation of corrected fluorescence intensity of SP bound to [file 12915_2022_1465_MOESM5_ESM.jpg]

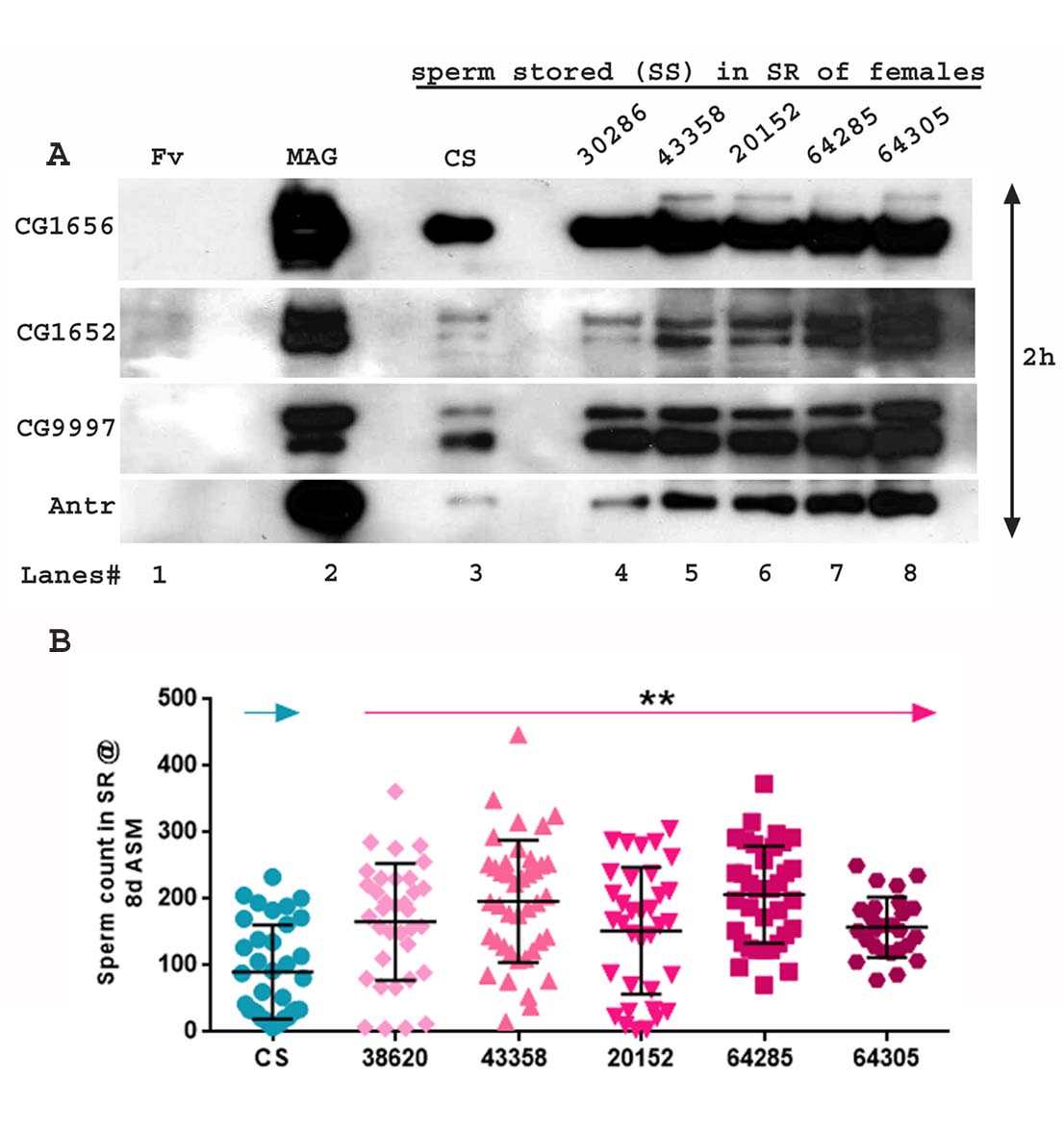

Supplement: Supplementary file 6 — Additional file 6: Figure S6. Hr39 mutant females have normal binding of LTR-SFPs to sperm but excessively retain sperm in storage. (A) Western blot probed for LTR-SFPs, at 2 h ASM. Lanes# 1: Fv, reproductive tract (RT) of 4 virgin females (negative control), 2: MAG, 1 pair of male accessory glands (positive control), 3: CS, sperm dissected from SR of 30 control (CS) females mated to wild type (CS) males, 4: 38620, sperm dissected from SR of 30 Hr39 mutant (BL38620) females mated to wild type (CS) males, 5: 43358, sperm dissected from SR of 30 Hr39 mutant (BL43358) females mated to wild type (CS) males, 6: 20152, sperm dissected from SR of 30 Hr39 mutant (BL20152) females mated to wild type (CS) males, 7: 64285, sperm dissected from SR of 30 Hr39 mutant (BL64285) females mated to wild type (CS) males, 8: 64305, sperm dissected from SR of 30 Hr39 mutant (BL64305) females mated to wild type (CS) males. Lanes were probed for LTR- SFPs , CG1656, CG1652, Antares and CG9997 as described in the text. (B) Graphical representation of sperm counts in SRs of CS and Hr39 females from all the five mutant stocks, mated to control ProtB-eGFP males (with eGFP tagged sperm; green; error bars show mean±SE; p**=<0.01; n=15-20) and frozen at 8 days ASM. [file 12915_2022_1465_MOESM6_ESM.jpg]

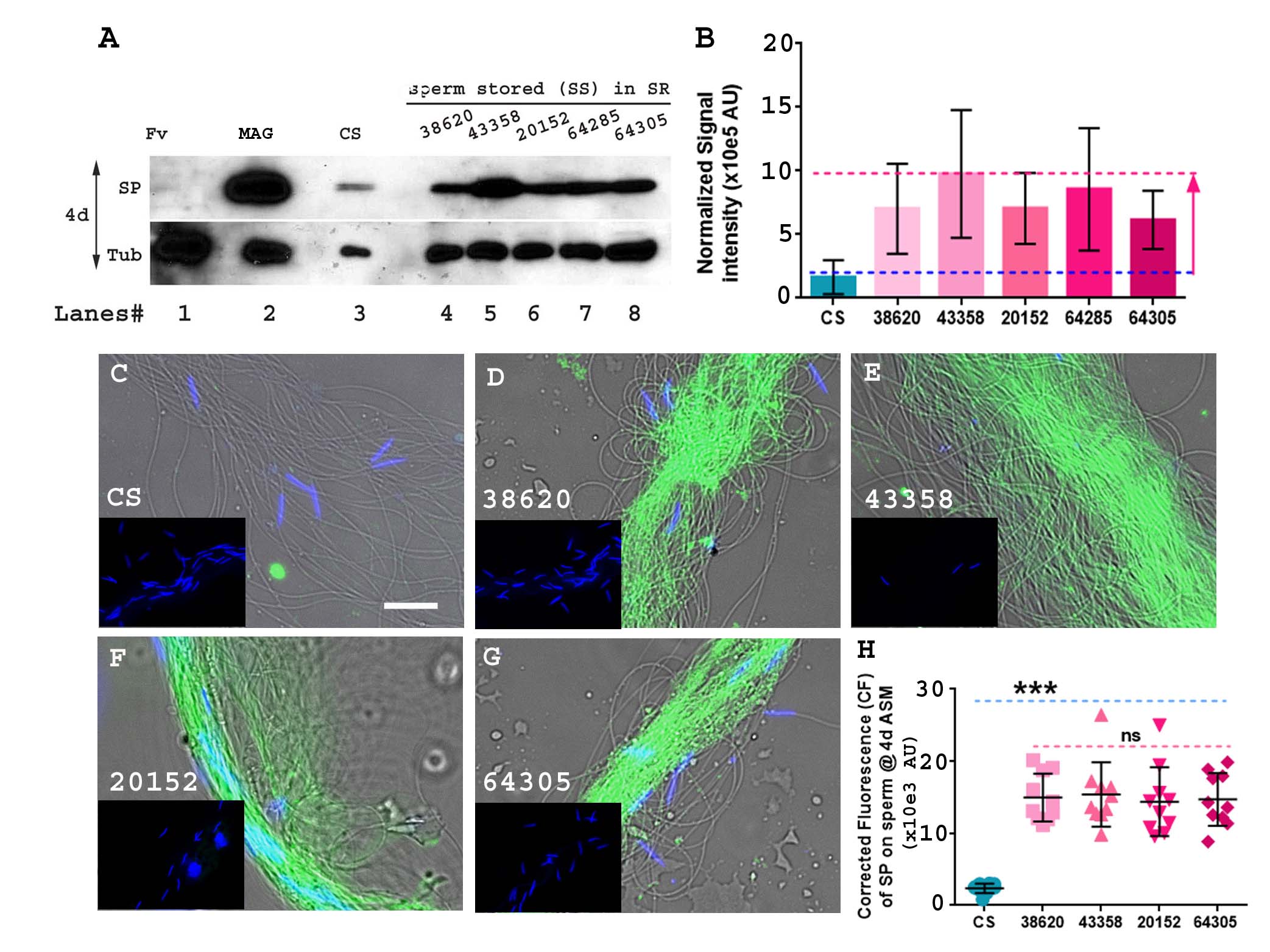

Supplement: Supplementary file 7 — Additional file 7: Figure S7. SP levels on sperm after normalization of Western blot and anti-SP staining on sperm dissected from Hr39 mutant females (with ablated SSCs and parovaria) show higher levels of SP levels when compared to their levels in sib-control or CS females at 4 days ASM. (A) Western blot probed for SP at 4 days ASM. Lanes# 1: Fv, reproductive tract (RT) of 4 unmated females (negative control), 2: MAG, 1 pair of male accessory glands (positive control), 3: CS, sperm dissected from SR of 30 control (CS) females mated to wild type (CS) males, 4: 38620, sperm dissected from SR of 30 Hr39 mutant (BL38620) females mated to wild type (CS) males, 5: 43358, sperm dissected from SR of 30 Hr39 mutant (BL43358) females mated to wild type (CS) males, 6: 20152, sperm dissected from SR of 30 Hr39 mutant (BL20152) females mated to wild type (CS) males, 7: 64285, sperm dissected from SR of 30 Hr39 mutant (BL64285) females mated to wild type (CS) males, 8: 64305, sperm dissected from SR of 30 Hr39 mutant (BL64305) females mated to wild type (CS) males. Tubulin (Tub) served as the loading control. (B) Graphical representation of the normalized levels of sperm bound SP in Hr39 mutant (red bars) females from all the five stocks relative to CS females (blue bar & dotted line) at 4 days ASM, as seen on one of three replicate Western blots; the other two blots showed similar results. Sperm samples isolated from the seminal receptacle of (C) CS (control) females and the other four Hr39 mutant females, (D) BL38620, (E) BL43358, (F) BL20152, (G) BL64305. The females were mated with CS males and frozen at 4 days ASM. Sperm heads were stained with DAPI (blue) and anti-SP staining was visualized with Alexa fluor 488, staining the sperm tail (green) and sperm head (cyan; overlapping blue/green). The insets show the respective negative controls for their panels. The larger panels have transmitted light filter added to show the outline of sperm tail in the regions where SP was und [file 12915_2022_1465_MOESM7_ESM.jpg]

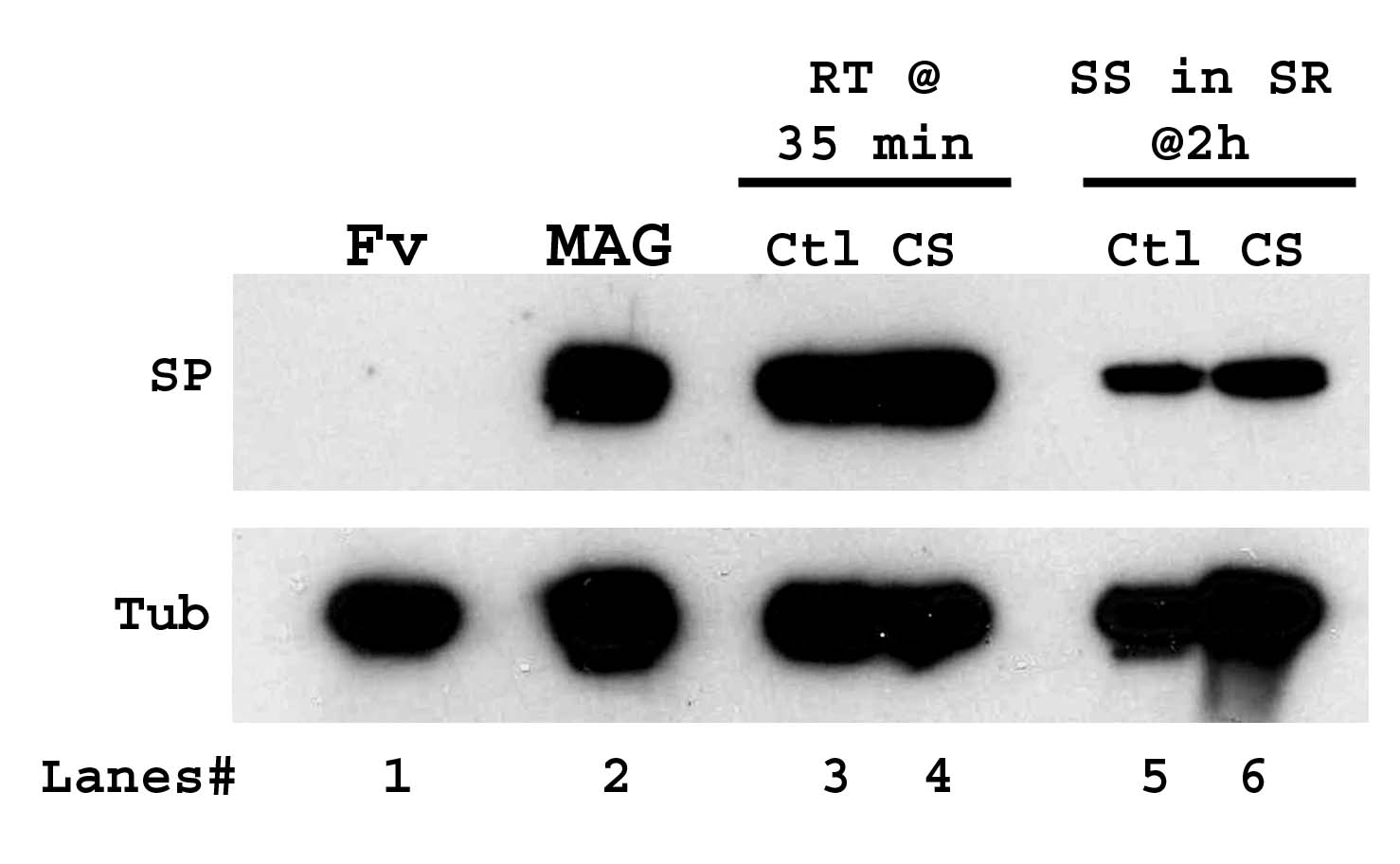

Supplement: Supplementary file 8 — Additional file 8: Figure S8. Levels of SP transferred to the FRT and bound to sperm stored in SR of balancer-sib control for BL64285 and CS females, do not show any significant difference at 2 h ASM, suggesting no evident background effect. Western blot probed for SP Lanes# 1: Fv, reproductive tract (RT) of 4 unmated females (negative control), 2: MAG, 1 pair of male accessory glands (positive control), 3: Ctl, reproductive tract (RT) of 4 balancer-sib (BL64285 {Hr39[C105]/CyO) control females mated to wild type (CS) males at 35 min ASM, 4: CS, RT of 4 CS females mated to wild type (CS) males at 35 min ASM, 5: Ctl sperm dissected from SR of 30 balancer-sib control females mated to wild type (CS) males, at 2 h ASM, 6: CS, sperm dissected from SR of 30 CS females mated to wild type (CS) males at 2 h ASM. Tubulin (Tub) served as the loading control. [file 12915_2022_1465_MOESM8_ESM.jpg]
