# Supplementary material for: Female factors modulate Sex Peptide’s association with sperm in Drosophila melanogaster
Source: BMC Biol. 2022 Dec 14;20:279. doi: 10.1186/s12915-022-01465-2 (PMC9749180; doi:10.1186/s12915-022-01465-2)
Supplement: Supplementary file 9 — Additional file 9. Uncropped blots. [file 12915_2022_1465_MOESM9_ESM.pdf]

Female factors modulate Sex Peptide's association with sperm in *Drosophila melanogaster*

Snigdha Misra, Norene A. Buehner, Akanksha Singh[1], Mariana F. Wolfner*

Department of Molecular Biology and Genetics, Cornell University, Ithaca NY-14853, USA

*Corresponding author
Email: mfw5@cornell.edu

[1]Present addresses:
SM: School of Health Sciences and Technology, University of Petroleum and Energy Studies, Dehradun, UK India
AS: Laboratory of System Genetics, National Heart Lung and Blood Institute, Bethesda, MD USA

Additional file 9: Uncropped blots

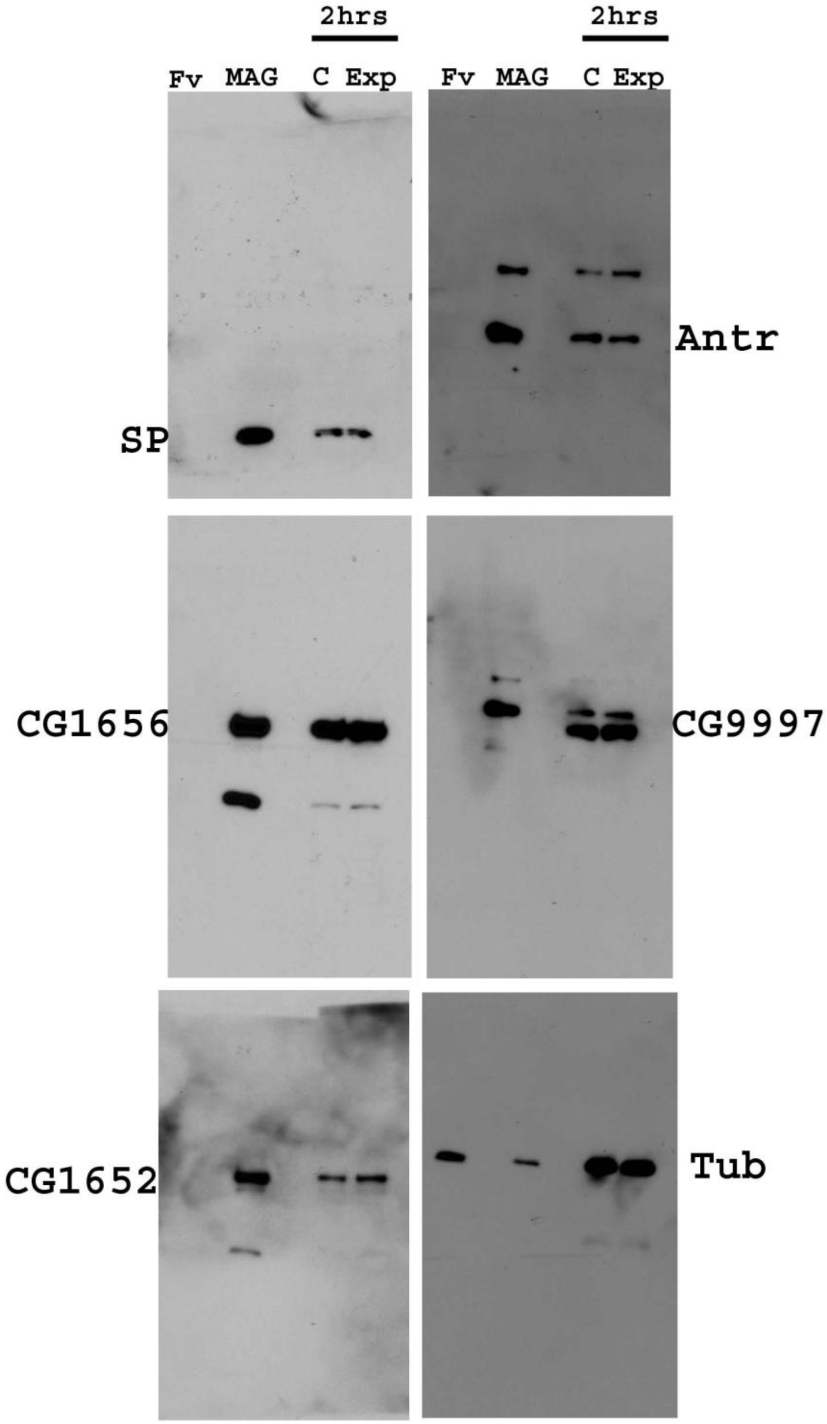

Unprocessed full length blots for **Figure. 3E** [SP, CG1656, CG1652, Antr, CG9997 and Tubulin (Tub)]. Occasionally in long-exposures such as shown here, minor cross-reactive bands are seen on Western blots with anti-Antr, CG1656 and CG 1652, as seen in some of the blots here; these bands were not included in the quantification reported in the paper. Immunofluorescence

experiments with knockout mutants also indicate that those cross-reactive bands do not detectably contribute to immunofluorescence signal [1].

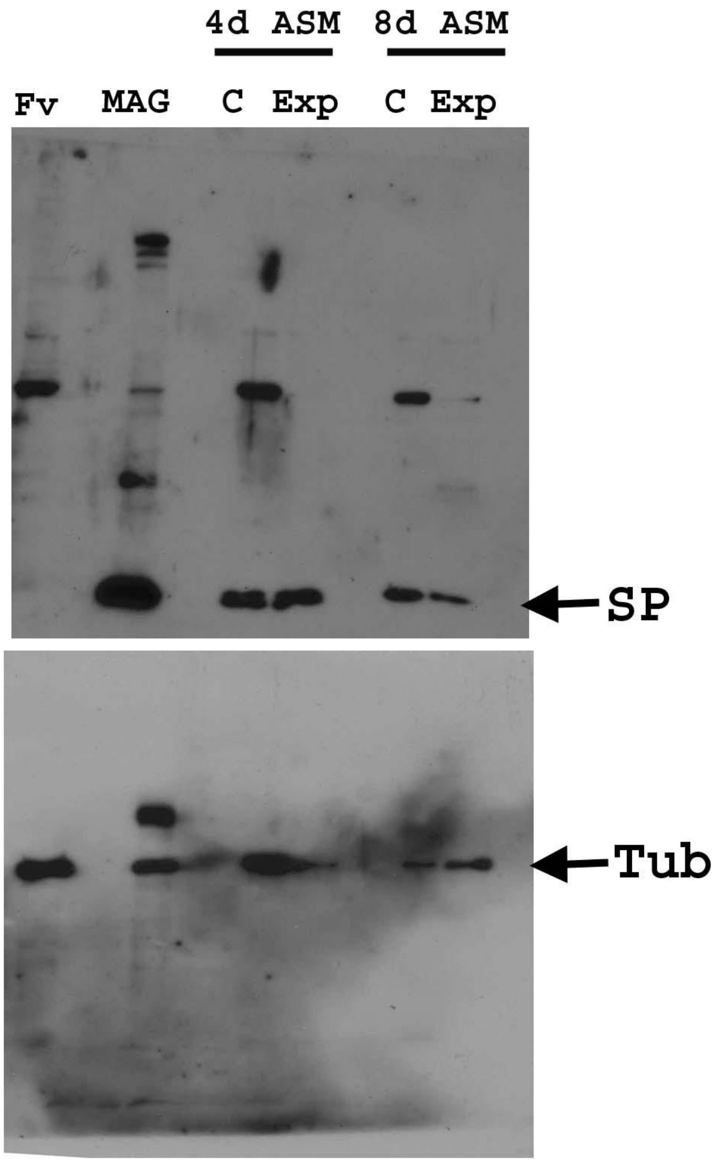

Unprocessed full length blots for **Figure. 3F** (SP and tubulin). In long exposures (such as shown here, high-molecular weight cross reactive bands are occasionally seen with these antibodies. Experiments with SP null mutants show that these bands are not SP, and do not contribute to the signal seen in immunofluorescence. The cross-reactive bands were not included in the quantification of Western blot signals reported in the paper.

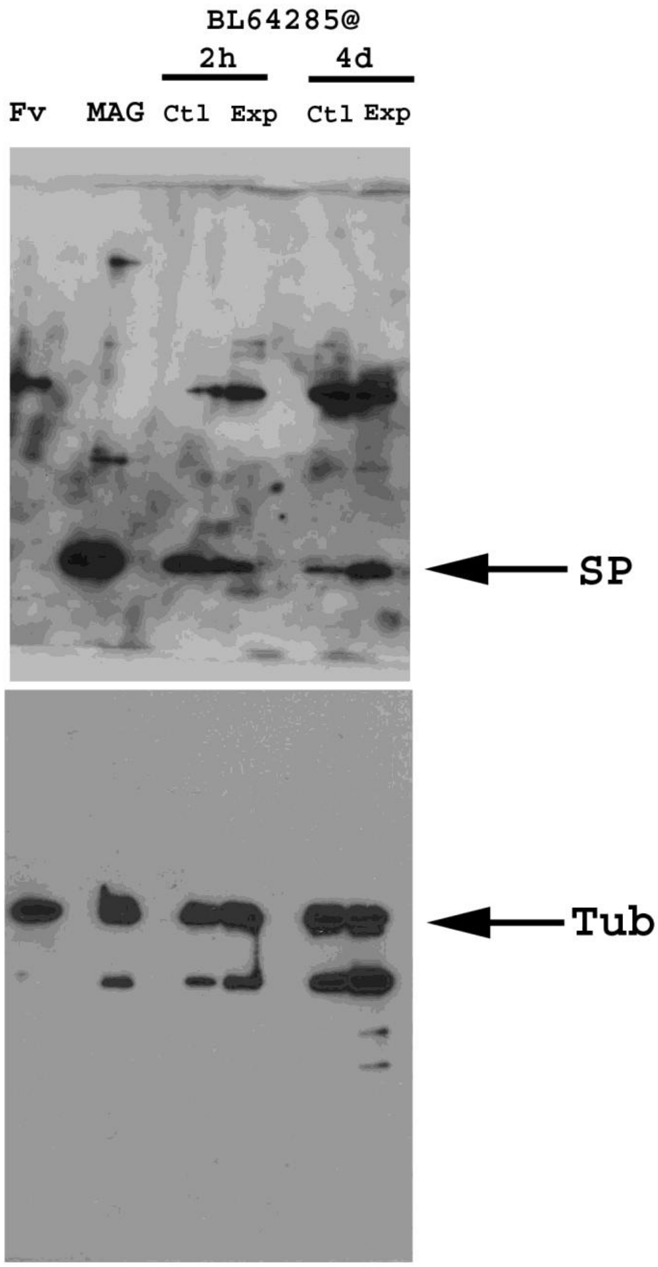

Unprocessed full length blots for **Figure. 4A** (SP and tubulin). In long exposures (such as shown here, high-molecular weight cross reactive bands are occasionally seen with these antibodies. Experiments with SP null mutants show that these bands are not SP, and do not contribute to the signal seen in immunofluorescence. The cross-reactive bands were not included in the quantification of Western blot signals reported in the paper.

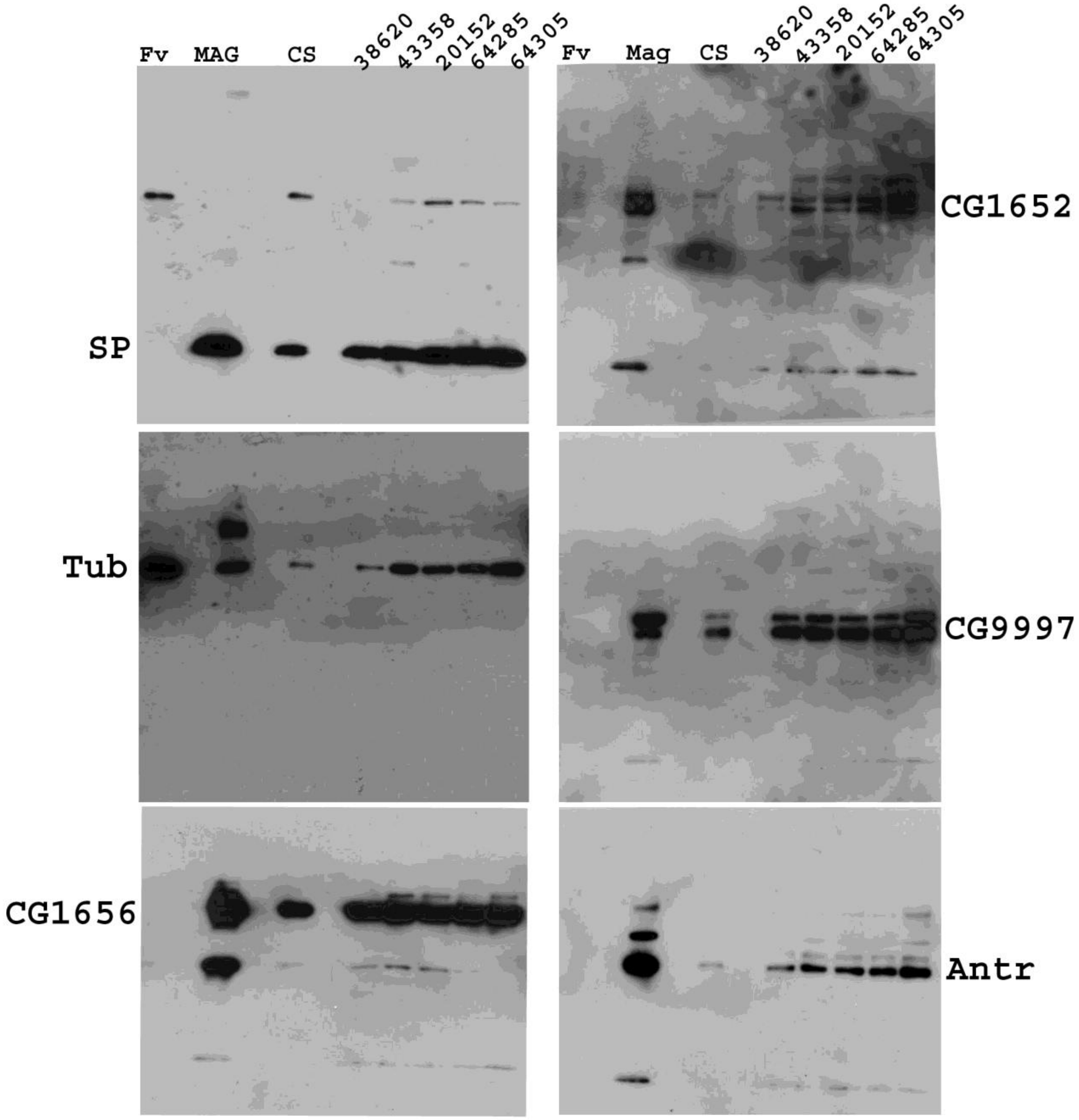

Unprocessed full length blots for **Additional file 5 Fig S5** (SP and Tubulin) **Additional file 6 Fig S6** (LTR-SFPs: CG1656, CG1652, CG9997 and Antares). Occasionally in long-exposures such as shown here, minor cross-reactive bands are seen on Western blots for tubulin and these SFPs, as seen in some of the blots here; these bands were not included in the quantification reported in the paper. Immunofluorescence experiments with knockout mutants also indicate that those cross-reactive bands do not detectably contribute to immunofluorescence signal [1].

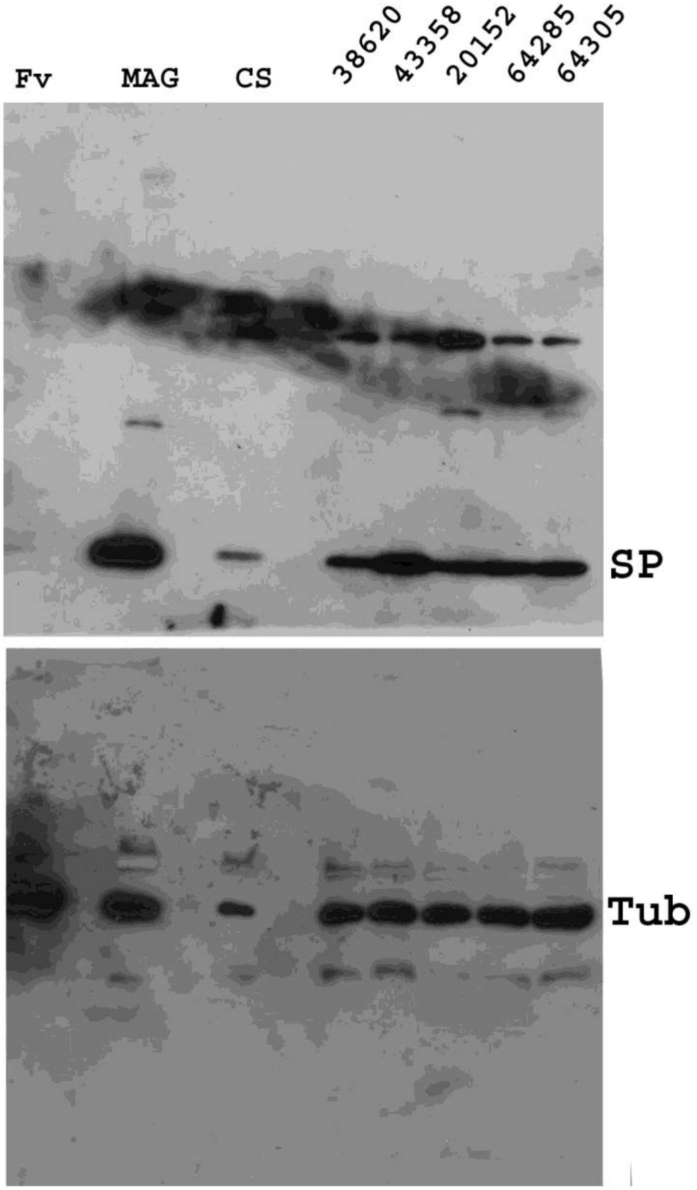

Unprocessed full length blots for **Additional file 7 Fig S7** (SP and tubulin). In long exposures (such as shown here, high-molecular weight cross reactive bands are occasionally seen with these antibodies. Experiments with SP null mutants show that these bands are not SP, and do not contribute to the signal seen in immunofluorescence [1]. The cross-reactive bands were not included in the quantification of Western blot signals reported in the paper.

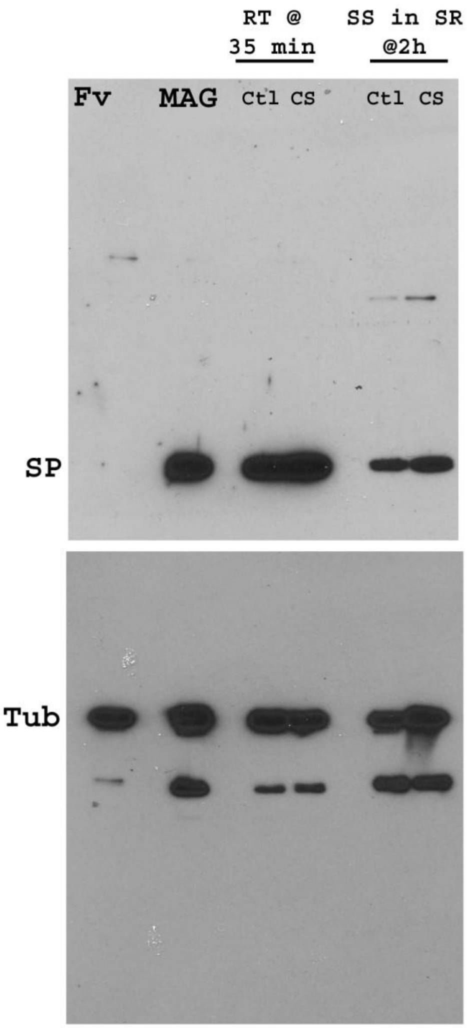

Unprocessed full length blots for **Additional file 8 Fig S8** (SP and tubulin). In long exposures (such as shown here, high-molecular weight cross reactive bands are occasionally seen with SP antibodies. Experiments with SP null mutants show that these bands are not SP, and do not contribute to the signal seen in immunofluorescence [1]. The cross-reactive bands were not included in the quantification of Western blot signals reported in the paper.

1. Singh A, Buehner NA, Lin H, Baranowski KJ, Findlay GD, Wolfner MF. Long-term interaction between *Drosophila* sperm and sex peptide is mediated by other seminal proteins that bind only transiently to sperm. Insect Biochem Mol Biol. 2018;102 September:43–51. doi:10.1016/j.ibmb.2018.09.004.
